# RNAi Regulator C3PO Promotes Arbovirus Infection in Insect Vectors

**DOI:** 10.1002/advs.202515869

**Published:** 2025-10-31

**Authors:** Yan Xiao, Tianyu Guan, Qianfeng Xia, Chen Chen, Qian Wang, Lan Luo, Hong Lu, Feng Cui

**Affiliations:** ^1^ State Key Laboratory of Animal Biodiversity Conservation and Integrated Pest Management Institute of Zoology Chinese Academy of Sciences Beijing 100101 China; ^2^ NHC Key Laboratory of Tropical Disease Control School of Tropical Medicine Hainan Medical University Haikou Hainan 570102 China; ^3^ University of Chinese Academy of Sciences Beijing 100049 China

**Keywords:** C3PO, miRNA precursor degradation, NHLRC2, planthopper, rice stripe virus, ROS

## Abstract

Numerous plant and human viruses depend on arthropods for their transmission. RNA interference (RNAi) constitutes a pivotal antiviral immune system in arthropods. Component 3 promoter of RISC (C3PO) complex plays an important regulatory role in small RNA production of the RNAi pathway. However, how C3PO affects viral infection in arthropod vectors remains elusive. Here, using the system of rice stripe virus (RSV) and its insect vector small brown planthopper, we found that C3PO facilitates RSV replication in insect vectors. C3PO directly degrades precursors of miRNAs, especially miR‐971‐3p. The RNA‐dependent RNA polymerase (RdRp) of RSV synergistically cooperates with C3PO to degrade miR‐971‐3p precursor. The reduction of miR‐971‐3p elevates the expression of target gene NHL repeat‐containing protein 2 (*NHLRC2*), which scavenges reactive oxygen species, thereby facilitating viral replication. Similar function of C3PO is elucidated in mosquitoes during the infection of sindbis virus, indicating the conservation of insect C3PO taking part in arbovirus infection. Our study extends the understanding of C3PO in insect vectors and offers the potential of C3PO as target for reducing arbovirus transmission.

## Graphical abstract

RNA interference (RNAi) is vital for eukaryotes to defend against virus infection. The C3PO complex takes part in RNAi but its role in regulating viral infection remains unclear. This work finds that insect C3PO facilitates arbovirus replication by degrading precursors of miRNAs, especially miR‐971‐3p. The reduction of miR‐971‐3p activates *NHLRC2* to scavenge reactive oxygen species induced by viral infection.

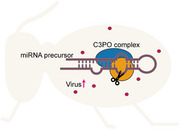

## 1 Introduction

Many plant and human viruses rely on arthropods, especially insects, for their transmission. These arthropod‐borne viruses (arboviruses) have posed substantial threats to public health and agricultural yield worldwide.^[^ ^1^, ^2^ ^]^ The propagative arboviruses maintain a balanced viral load within their insect vectors to optimize both vector health and viral transmission efficiency. Insect innate immune systems interact with arboviruses to finely regulate viral replication levels. RNA interference (RNAi) pathway constitutes one of the most essential antiviral immune systems in invertebrates, plants and mammals.^[^ ^3^, ^4^, ^5^ ^]^ Two types of small RNAs, the small interfering RNA (siRNA) and microRNA (miRNA), are produced from the RNAi pathway and function with different mechanisms. siRNAs, derived from viral double‐stranded RNAs (dsRNAs), exhibit full complementarity to the viral genome, leading to a powerful antiviral effect.^[^ ^6^, ^7^ ^]^ Conversely, miRNAs are integral to the post‐transcriptional regulation of host or viral genes through incomplete base pairing, producing antiviral outcomes or promoting viral infection.^[^ ^8^, ^9^, ^10^, ^11^, ^12^ ^]^

In addition to the key components such as Drosha, Pasha, Dicer, and Argonaute (Ago), Component 3 promoter of RISC (C3PO) plays an important regulatory role in the RNAi pathway.^[^ ^13^, ^14^ ^]^ C3PO consists of Translin and Translin‐associated factor X (Trax) in a 6:2 ratio with an asymmetric octameric barrel structure.^[^ ^15^ ^]^ Translin acts as a DNA/RNA‐binding protein and Trax is responsible for nucleic acid endonuclease activity.^[^ ^16^ ^]^ C3PO participates in miRNA and siRNA processing with an opposite consequence. C3PO inhibits miRNA production through degrading miRNA precursors with mismatched bulges in the stem region^[^ ^14^ ^]^ while it promotes siRNA functions by assisting Ago2 to degrade the passenger strands of siRNA duplexes.^[^ ^13^ ^]^ Although C3PO has been reported to play crucial roles in numerous biological processes in animals such as cell proliferation, maintenance of telomere‐related transcript homeostasis, hippocampal synaptic plasticity, and pathogenic vascular stiffness,^[^ ^17^, ^18^, ^19^, ^20^ ^]^ whether C3PO affects viral infection in arthropod vectors through regulating RNAi molecules remains elusive.

Rice stripe virus (RSV), a nonenveloped negative‐strand RNA virus belonging to the *Tenuivirus* genus, causes one of the most detrimental rice stripe diseases in temperate and subtropical regions.^[^ ^21^ ^]^ The RSV genome consists of four single‐stranded RNA segments encoding one nucleocapsid protein (NP), one RNA‐dependent RNA polymerase (RdRp), and five nonstructural proteins.^[^ ^21^, ^22^ ^]^ RSV is efficiently transmitted by the small brown planthopper *Laodelphax striatellus* and is capable of proliferating in midgut epithelial cells.^[^ ^1^ ^]^ Viral replication level is under the control of insect immune systems. Apart from apoptosis and prophenoloxidase activation pathway,^[^ ^23^, ^24^, ^25^ ^]^ RNAi contributes a lot to maintain a limited RSV titer within insect vectors.^[^ ^26^, ^27^ ^]^ Our previous study demonstrated a potential interplay between RSV and the C3PO complex of planthoppers. RSV RdRp bound Translin and interference of *Translin* expression inhibited RSV amounts,^[^ ^28^ ^]^ indicating a positive role of C3PO for RSV infection.^[^ ^28^ ^]^ Based on the contrary action of C3PO on miRNA and siRNA processing, we speculate that the promotion of RSV replication relies on the inhibition of miRNA production by C3PO. The C3PO‐degraded miRNAs and their roles in viral infection have not been elucidated.

In this study, we explored the mechanisms by which C3PO facilitates arbovirus replication in insect vectors. Based on gene interference combined with small RNA‐seq and miRNA precursor degradation experiments, we screened miRNAs that were directly processed by C3PO in small brown planthoppers. The roles of one key miRNA as well as its target gene in controlling RSV replication were deeply elucidated. CRISPR‐Cas9‐generated *Translin* mutants of planthoppers were applied to evaluate the comprehensive effects of C3PO on RSV replication and the following transmission. The functional conservation of C3PO in promoting arbovirus infection was assessed in two pairs of arthropods along with their transmitted human viruses.

## 2 Results

### 2.1 miRNAs Regulated by C3PO and Responding to RSV Infection in Planthoppers

**Figure 1:**
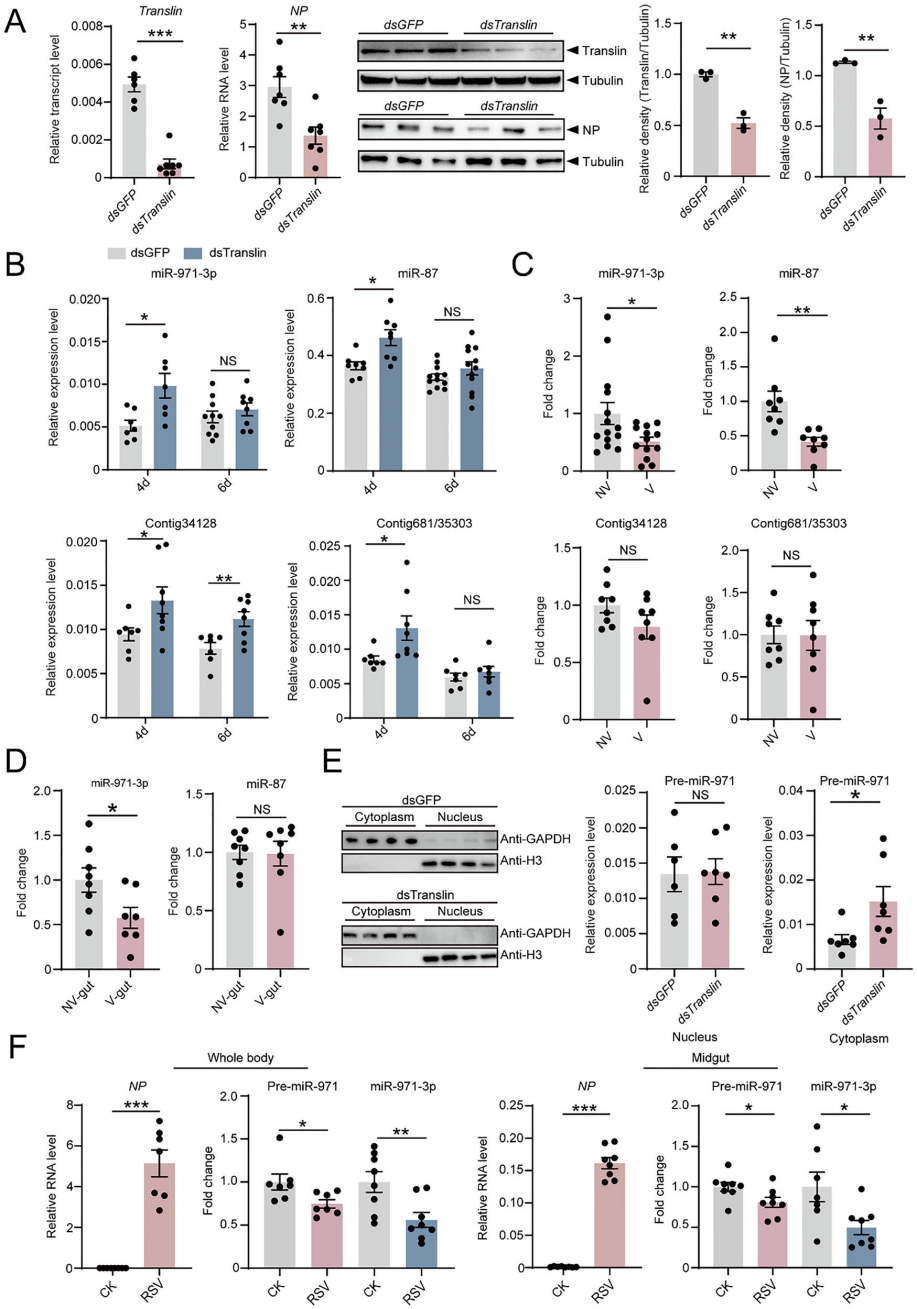
miRNAs regulated by C3PO and responding to RSV infection in planthoppers. A) The transcript levels of *Translin* and the RNA levels of RSV *NP* relative to that of *EF2* of viruliferous planthoppers at 6 days after injection of ds*Translin*‐RNA or ds*GFP*‐RNA with qPCR assays. n = 6–7 for each group. Protein levels of Translin or RSV NP were analyzed with western blot assays using an anti‐Translin polyclonal antibody or anti‐NP monoclonal antibody. *β*‐tubulin was measured as internal control using an anti‐*β*‐tubulin monoclonal antibody. The grayscale of NP relative to that of *β*‐tubulin is compared between groups. n = 3 for each group. B) Verification of expression levels of miR‐971‐3p, miR‐87, Contig34128 and Contig681/35303 relative to that of *U6* using qPCR. n = 7–12 for each group. C) Fold change of miR‐971‐3p, miR‐87, Contig34128 and Contig681/35303 in the whole bodies of viruliferous (V) planthoppers compared to those in nonviruliferous (NV) planthoppers measured by qPCR. n = 8–14 for each group. D) Fold change of miR‐971‐3p and miR‐87 in the midguts of viruliferous (V) planthoppers compared to those in nonviruliferous (NV) planthoppers measured by qPCR. n = 7–8 for each group. E) Expression levels of pre‐miR‐971 relative to that of *U6* in the cytoplasm and nuclear extracts of viruliferous planthoppers at 4 days after injection of ds*Translin‐*RNA or ds*GFP‐*RNA, as assessed by qPCR. n = 6–7 for each group. For the detection of nuclear and cytoplasmic proteins, histone H3 and GAPDH were used as markers, respectively, with monoclonal anti‐H3 antibody and polyclonal anti‐GAPDH antibody. F) The RNA levels of RSV *NP* and fold change of miR‐971‐3p or its precursor in the whole bodies and midguts of planthoppers at 8 days after injection of RSV crude extracts compared to those of injection with crude extracts from nonviruliferous planthoppers (CK). n = 7–8 for each group. The values are presented as mean ± SE. Differences were statistically evaluated using Student's *t*‐test for comparison between two groups. *, *p* < 0.05. **, *p* < 0.01.***, *p* < 0.001. NS, no significant differences.

In the viruliferous planthoppers, we injected double‐stranded RNA of *Translin* (ds*Translin*‐RNA), *Trax* (ds*Trax*‐RNA) or *GFP* (ds*GFP*‐RNA). The knockdown of *Translin* expression resulted in an obvious reduction of RSV accumulation at 6 days post inoculation (dpi) in terms of the RNA and protein levels of RSV *NP* (**Figure** **1**A), while *Trax* knockdown did not produce substantial influence on RSV amount (Figure S1, Supporting Information), indicating that Translin played a predominant role in facilitating RSV infection. To screen C3PO‐processed miRNAs, small RNA‐seq was applied in viruliferous planthoppers at 4 and 6 dpi with injection of ds*Translin*‐RNA or ds*GFP*‐RNA with two replicates for each group. More than 25M clean reads were obtained for each sample (GenBank PRJNA1133975) and mapped to the genome of small brown planthopper.^[^ ^29^ ^]^ The size of planthopper sRNAs ranged from 18‐nt to 30‐nt with a peak at 22‐nt (Figure S2, Supporting Information). Totally 443 miRNAs were detected for expression including novel miRNAs (Table S1, Supporting Information). Seventeen miRNAs displayed differential expression between ds*Translin*‐RNA and ds*GFP*‐RNA group with a threshold of more than twofold difference and an adjusted *p* value lower than 0.05, including eight upregulated and nine downregulated miRNAs in the *Translin*‐knockdown group (Table S2, Supporting Information). However, one of the upregulated miRNAs, novel_mir58, was not successfully cloned and sequenced, and excluded from further study. Considering that the upregulated miRNAs correlated with C3PO dysfunction, we quantified their expression using real‐time quantitative PCR (qPCR). Only miR‐971‐3p, miR‐87 and two novel miRNAs (Contig34128 and Contig681/35303) exhibited elevated expression when *Translin* was knocked down (Figure 1B), while the expression of the remaining three miRNAs did not increase (Figure S3, Supporting Information).

The response of the four miRNAs to RSV infection in planthoppers was further explored with qPCR assays. miR‐971‐3p and miR‐87 had lower expression levels in the whole body of viruliferous planthoppers compared to nonviruliferous planthoppers and no variation was for Contig34128 and Contig681/35303 (Figure 1C). Especially in the midgut, the major organ where RSV replicates, only miR‐971‐3p displayed a lower expression (Figure 1D). Following *Translin* knockdown in viruliferous planthoppers, the expression level of miR‐971‐3p precursor (pre‐miR‐971) significantly increased in the cytoplasm, but not in the nucleus, in accordance with the elevated miR‐971‐3p (Figure 1E). When nonviruliferous planthoppers were injected with RSV crude extracts, pre‐miR‐971 and miR‐971‐3p were significantly decreased in the whole body and midgut at 8 dpi (Figure 1F). Consequently, miR‐971‐3p could be the target regulated most significantly by C3PO in planthoppers.

### 2.2 RSV Collaborates with C3PO to Degrade miR‐971‐3p Precursor

**Figure 2:**
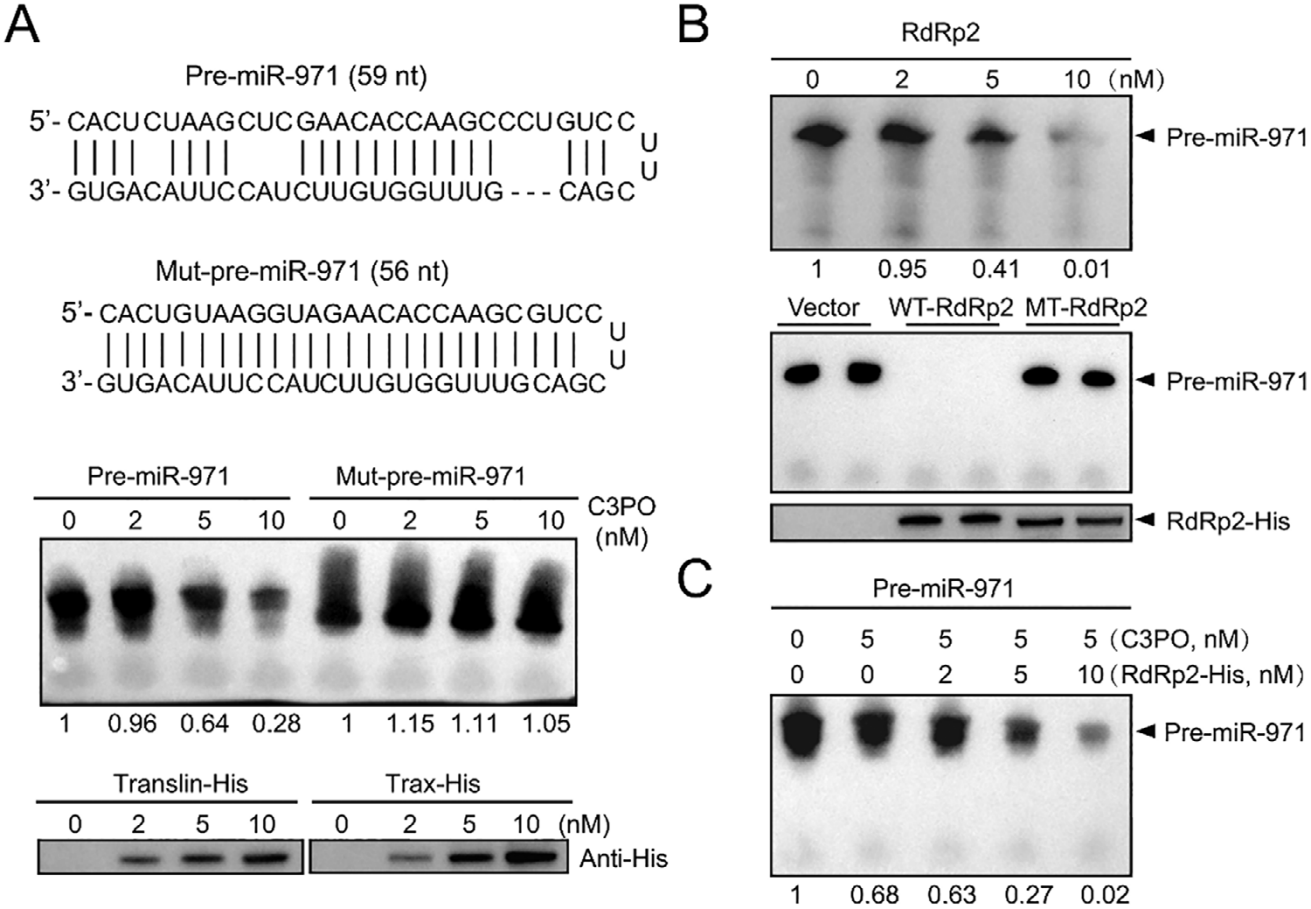
RSV collaborates with C3PO to degrade miR‐971‐3p precursor. A) The direct degradation assay of C3PO complex to precursors of miR‐971‐3p and mutants (Mut) labeled with biotin. Different concentrations of C3PO (Translin‐His and Trax‐His) are shown in western blot assay using an anti‐His monoclonal antibody. B) Degradation assay of biotin‐labeled miR‐971‐3p precursor in the presence of different concentrations of RSV RdRp2‐His, wild‐type (WT) or mutant (MT) RdRp2. The WT‐ and MT‐RdRp2 are shown in western blot using an anti‐His monoclonal antibody. C) Degradation assay of C3PO to biotin‐labeled miR‐971‐3p precursor in the presence of different concentrations of RSV RdRp2‐His. For (A), (B) and (C) the relative grayscale values of miRNA precursors were shown underneath the photo. The purified products from pET28a vector serve as negative control.

C3PO is supposed to degrade miRNA precursors by cleaving mismatched bulges within their stem regions.^[^ ^14^ ^]^ The secondary structure of pre‐miR‐971 had mismatched bulges in the stem region (**Figure** **2**A). We expressed and purified recombinant Translin‐His and Trax‐His proteins to verify the direct degradation of C3PO complex to the 5′ biotin‐labeled pre‐miR‐971 in vitro. The putative molecular weights of Translin (gene set evm.model.Contig223.14) and Trax (gene set evm.model.Contig256.29) of small brown planthopper were 26.8 and 37.2 kD. An increase in C3PO concentration resulted in enhanced degradation of pre‐miR‐971, with only 43% remaining at 10 nM C3PO, while C3PO did not degrade the pre‐miR‐971 mutant without mismatched bulges in stem regions (Figure 2A, Figure S4, Supporting Information). In previous work, we found that the fragment of RSV RdRp from 482 to 1051 amino acid residues (RdRp2) bound Translin.^[^ ^28^ ^]^ Considering that RdRp possesses endonuclease activities on single strand RNA and the RdRp2 fragment contains four endonuclease active sites (D547, D567, E585 and K604),^[^ ^30^ ^]^ we assessed the cleavage of pre‐miR‐971 by RdRp2‐His. With the increase of RdRp2‐His, the degradation of pre‐miR‐971 was enhanced (Figure 2B). Mutations at the four endonuclease active sites abolished this degradation capacity (Figure 2B). The degradation of pre‐miR‐971 was more pronounced in the presence of both C3PO and RdRp2‐His (Figure 2C). These findings indicated that RSV not only binds C3PO but also synergistically cooperates with C3PO to degrade miR‐971‐3p precursor.

### 2.3 miR‐971‐3p Inhibits RSV Replication by Targeting *NHLRC2*

**Figure 3:**
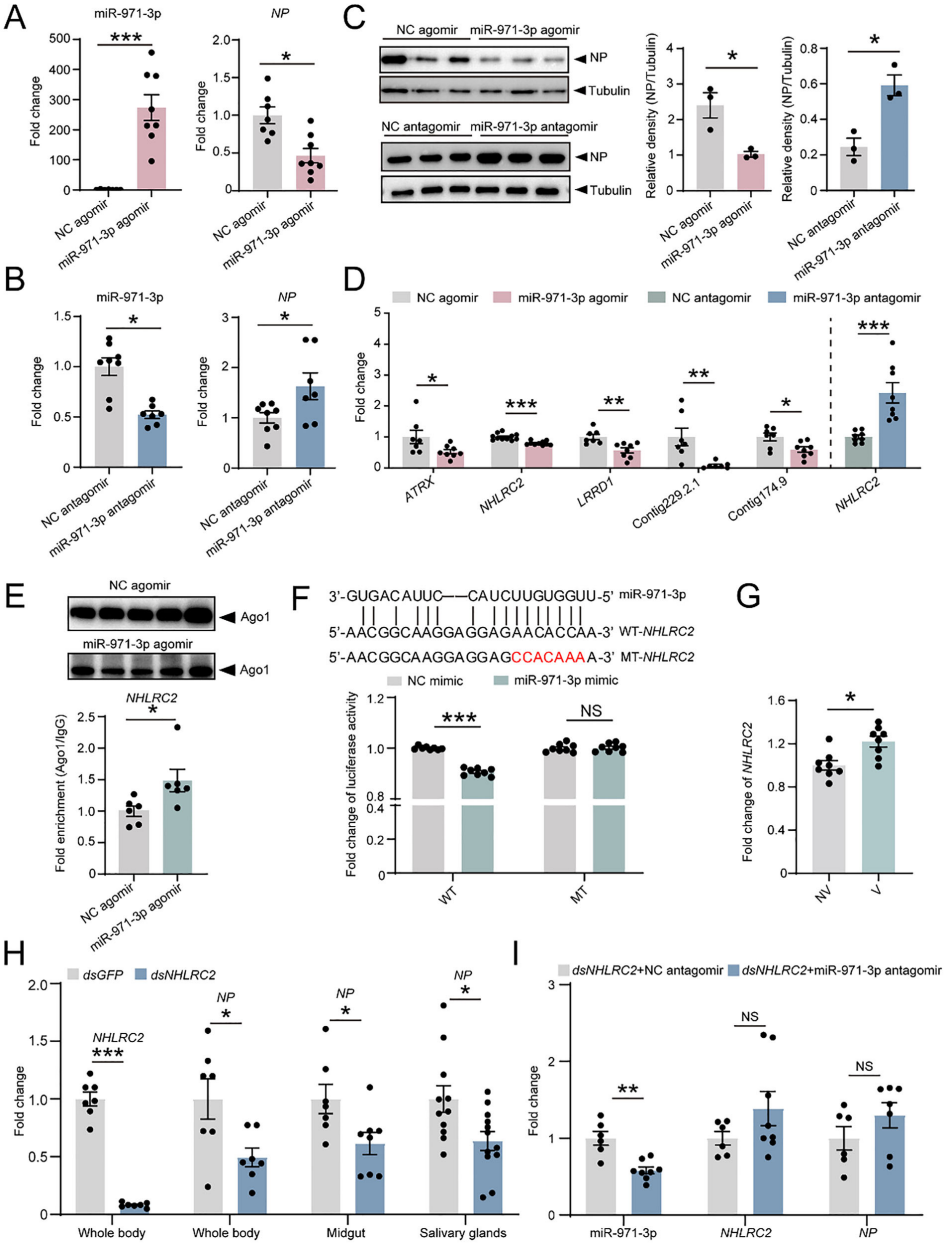
miR‐971‐3p inhibits RSV replication by targeting *NHLRC2*. A,B) Fold change of miR‐971‐3p and RNA levels of RSV *NP* in viruliferous planthoppers at 6 days after injection of miR‐971‐3p agomir (A) or antagomir (B) compare to those of injection with NC agomir or NC antagomir. n = 7–8 for each group. C) Protein levels of RSV NP in the samples of (A) and (B) analyzed with western blot using an anti‐NP monoclonal antibody. *β*‐tubulin is measured as internal control using an anti‐*β*‐tubulin monoclonal antibody. The grayscale of NP relative to that of *β*‐tubulin is compared between groups. n = 3 for each group. D) Fold change of transcript levels of miR‐971‐3p putative target genes in nonviruliferous planthoppers at 6 days after injection of miR‐971‐3p agomir or antagomir compared to those of injection with NC agomir or NC antagomir. n = 7–11 for each group. E) RIP‐qPCR assay on *NHLRC2* enriched in the Ago1‐immunoprecipitation from planthoppers injected with miR‐971‐3p agomir or NC agomir using a homemade anti‐Ago1 monoclonal antibody. Mouse IgG serves as negative control. The fold enrichment of *NHLRC2* in the Ago1‐immunoprecipitation relative to that in the IgG sample is compared. n = 6 for each group. The protein levels of Ago1 in the immunoprecipitations are presented in western blot. Different lanes illustrated the enrichment of Ago1 across various biological replicates in the planthoppers injected with NC agomir or miR‐971‐3p agomir. F) Dual luciferase assay on the interaction between miR‐971‐3p and wild‐type (WT) or mutant (MT) *NHLRC2* in the presence of 50 nM miR‐971‐3p mimic in *Drosophila* S2 cells. NC mimic is used as negative control. The Renilla luciferase activity relative to firefly luciferase activity in the negative control group is set as 1. n = 8 for each group. G) Fold change of *NHLRC2* transcript level in viruliferous (V) planthoppers compare to that in nonviruliferous (NV) planthoppers. n = 8 for each group H) Fold change of *NHLRC2* transcript levels and RNA levels of RSV *NP* in the whole bodies, midguts and salivary glands of planthoppers at 6 days after injection of ds*NHLRC2*‐RNA compared to those of injection with ds*GFP*‐RNA. n = 7–12 for each group. I) Fold change of miR‐971‐3p expression level, *NHLRC2* transcript levels and RSV *NP* RNA levels in planthoppers at 6 days after co‐injection of ds*NHLRC2*‐RNA and miR‐971‐3p antagomir compared to those of co‐injection with ds*NHLRC2*‐RNA and NC antagomir. n = 6–8 for each group. The values are presented as mean ± SE. Differences were statistically evaluated using Student's *t*‐test for comparison between two groups. *, *p* < 0.05. **, *p* < 0.01. ***, *p* < 0.001. NS, no significant differences.

When synthesized miR‐971‐3p agomir was injected in viruliferous planthoppers, the RNA and protein level of RSV *NP* significantly decreased at 6 dpi compared to the injection of negative control (NC) agomir (**Figure** **3**A,C). Conversely, reducing the expression of miR‐971‐3p with the injection of its antagomir made RSV amounts enhanced at 6 dpi (Figure 3B,C). Therefore, miR‐971‐3p had a negative impact on RSV replication.

Using both miRanda and RNAhybrid algorithms, a total of 212 genes were predicted as targets of miR‐971‐3p in planthoppers (Table S3, Supporting Information). The top 30 candidate genes were verified in nonviruliferous planthoppers with injection of miR‐971‐3p agomir or antagomir. The expression levels of five genes, encoding transcriptional regulator *ATRX*, NHL repeat‐containing protein 2 (*NHLRC2*), leucine‐rich repeat and death domain‐containing protein 1 (*LRRD1*), and two unknown proteins, significantly dropped down with injection of miR‐971‐3p agomir (Figure 3D), whereas other 25 genes were upregulated or kept unchanged (Figure S5A,B, Supporting Information). Among the five genes downregulated by miR‐971‐3p, only *NHLRC2* displayed elevated expression upon treatment with miR‐971‐3p antagomir (Figure 3D, Figure S5C, Supporting Information). Ago1 is an essential component for miRNA‐mediated target suppression in insects.^[^ ^31^ ^]^ RNA immunoprecipitation combined with qPCR (RIP‐qPCR) assay using a homemade anti‐Ago1 monoclonal antibody showed that *NHLRC2* was enriched in the Ago1‐immunoprecipitation from planthoppers injected with miR‐971‐3p agomir compared to the NC agomir injection group (Figure 3E). The direct interaction between miR‐971‐3p and a 300 bp segment of *NHLRC2* containing the putative target site was verified by dual luciferase assay in *Drosophila* S2 cells. Plasmids harboring *NHLRC2* target site displayed reduced luciferase activity in the presence of 50 nM miR‐971‐3p mimic, whereas the mutations in the target site eliminated miR‐971‐3p's suppressive effect (Figure 3F). These results demonstrated that miR‐971‐3p negatively regulated *NHLRC2* expression.

In viruliferous planthoppers, *NHLRC2* had a higher expression level than in nonviruliferous planthoppers (Figure 3G), corresponding to the reduced levels of miR‐971‐3p (Figure 1C). When *NHLRC2* was knocked down with injection of ds*NHLRC2*‐RNA, the RNA level of RSV *NP* significantly decreased in the whole bodies, midguts and salivary glands of planthoppers at 6 dpi (Figure 3H). In the rescue experiment where ds*NHLRC2*‐RNA and miR‐971‐3p antagomir were simultaneously injected, the transcript level of *NHLRC2* and the RNA level of RSV *NP* remained unchanged in the whole bodies, despite a reduction in miR‐971‐3p expression (Figure 3I). This indicated that *NHLRC2* plays a positive role in maintaining RSV amounts and is the primary downstream factor in the antiviral effects mediated by miR‐971‐3p.

### 2.4 NHLRC2 Scavenges ROS to Facilitate RSV Replication

**Figure 4:**
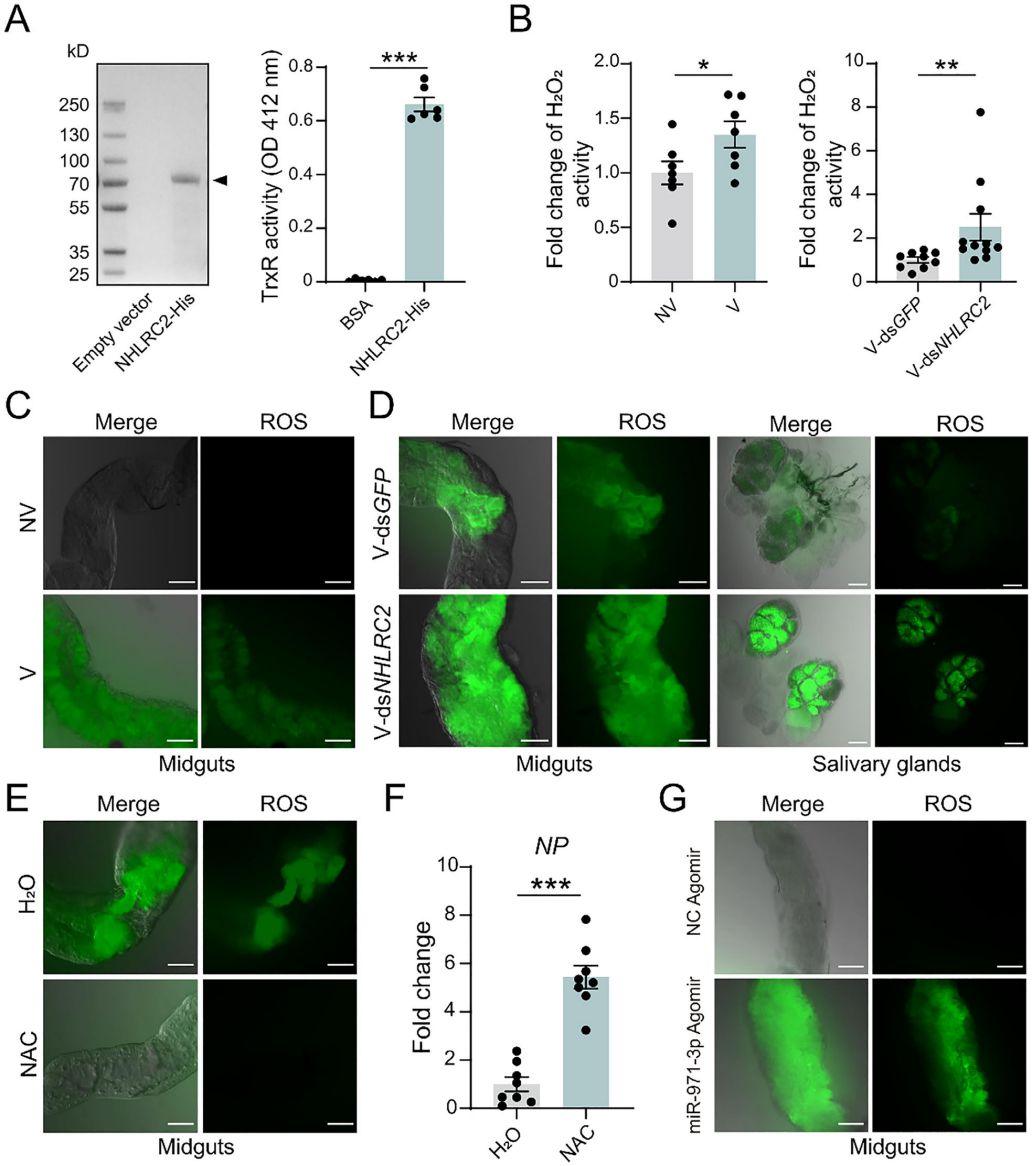
NHLRC2 scavenges ROS to facilitate RSV replication. A) Thioredoxin reductase (TrxR) assay on recombinant NHLRC2‐His. The purified NHLRC2‐His is shown in SDS‐PAGE. The OD 412 nm values reflecting TNB are used to show TrxR activity. BSA is used as negative control. n = 6 for each group B) Fold change of H_2_O_2_ activities in viruliferous (V) planthoppers compared to nonviruliferous (NV) planthoppers or in viruliferous planthoppers after injection of ds*NHLRC2*‐RNA compared to the injection of ds*GFP*‐RNA. n = 7–11 for each group. C) ROS activities in the midgut epithelial cells from nonviruliferous (NV) or viruliferous (V) planthoppers. D) ROS activities in midgut epithelial cells and salivary glands from viruliferous planthoppers at 6 days after injection of ds*NHLRC2*/ds*GFP*‐RNA. E) ROS activities in midgut epithelial cells of planthoppers fed on RSV‐infected rice for 6 days with the injection of the ROS inhibitor, N‐acetyl‐L‐cysteine (NAC). Control group is injected with H_2_O. F) Fold change of the RNA level of RSV *NP* in the samples of (E). n = 8 for each group G) ROS activities in midgut epithelial cells from nonviruliferous planthoppers after injection of miR‐971‐3p agomir/NC agomir. For (C) (D), (E) and (G), ROS activities were measured using a DCFH‐DA assay kit under a confocal microscope. Scale bars, 50 µm. “Merge” means the bright field image merging with the fluorescence signal. For (A), (B) and (F), the values are presented as mean ± SE. Differences were statistically evaluated using Student's *t*‐test for comparison between two groups. *, *p* < 0.05. **, *p* < 0.01. ***, *p* < 0.001.

*NHLRC2* (gene set number evm.model.Contig58.165) encodes a putative 74.6 kD protein that consists of a N‐terminal thioredoxin domain and a C‐terminal NHL‐repeat domain. The thioredoxin domain contains a CINC motif, which is characteristic of oxidoreductases and typically involved in thiol‐disulfide exchange.^[^ ^32^ ^]^ To investigate the potential activity of planthopper NHLRC2, the expressed and purified recombinant NHLRC2‐His was analyzed in a thioredoxin reductase assay, yielding an activity of 121.63 U mg^−1^ protein (**Figure** **4**A). Thioredoxin serves as a key component in a redox regulatory system and scavenges reactive oxygen species (ROS).^[^ ^33^ ^]^ In viruliferous planthoppers, RSV has achieved equilibrium between viral load and insect's immune systems. Compared to nonviruliferous insects, viruliferous planthoppers exhibited elevated H_2_O_2_ level in the whole bodies (Figure 4B) and enhanced ROS activity in the midgut epithelial cells (Figure 4C) as demonstrated by a DCFH‐DA assay kit under a confocal microscope. However, knockdown of *NHLRC2* expression via ds*NHLRC2*‐RNA injection significantly elevated H_2_O_2_ levels in the whole bodies (Figure 4B) and ROS activity in midguts and salivary glands of viruliferous planthoppers (Figure 4D), indicating that NHLRC2 was able to negatively regulate ROS. When the ROS inhibitor, N‐acetyl‐L‐cysteine (NAC), was injected in nonviruliferous planthoppers, which were then fed on RSV‐infected rice for 6 d, the RNA level of *NP* significantly increased with the elimination of ROS (Figure 4E,F). We further explored the effect of miR‐971 on the ROS activity in nonviruliferous planthoppers. The ROS activity in the midgut epithelial cells was enhanced with the injection of miR‐971‐3p agomir compared to the NC agomir injection group as demonstrated in a DCFH‐DA assay (Figure 4G). These results demonstrated that NHLRC2 promotes RSV replication through scavenging ROS.

### 2.5 The Comprehensive Effects of C3PO on RSV Replication and the Following Transmission

**Figure 5:**
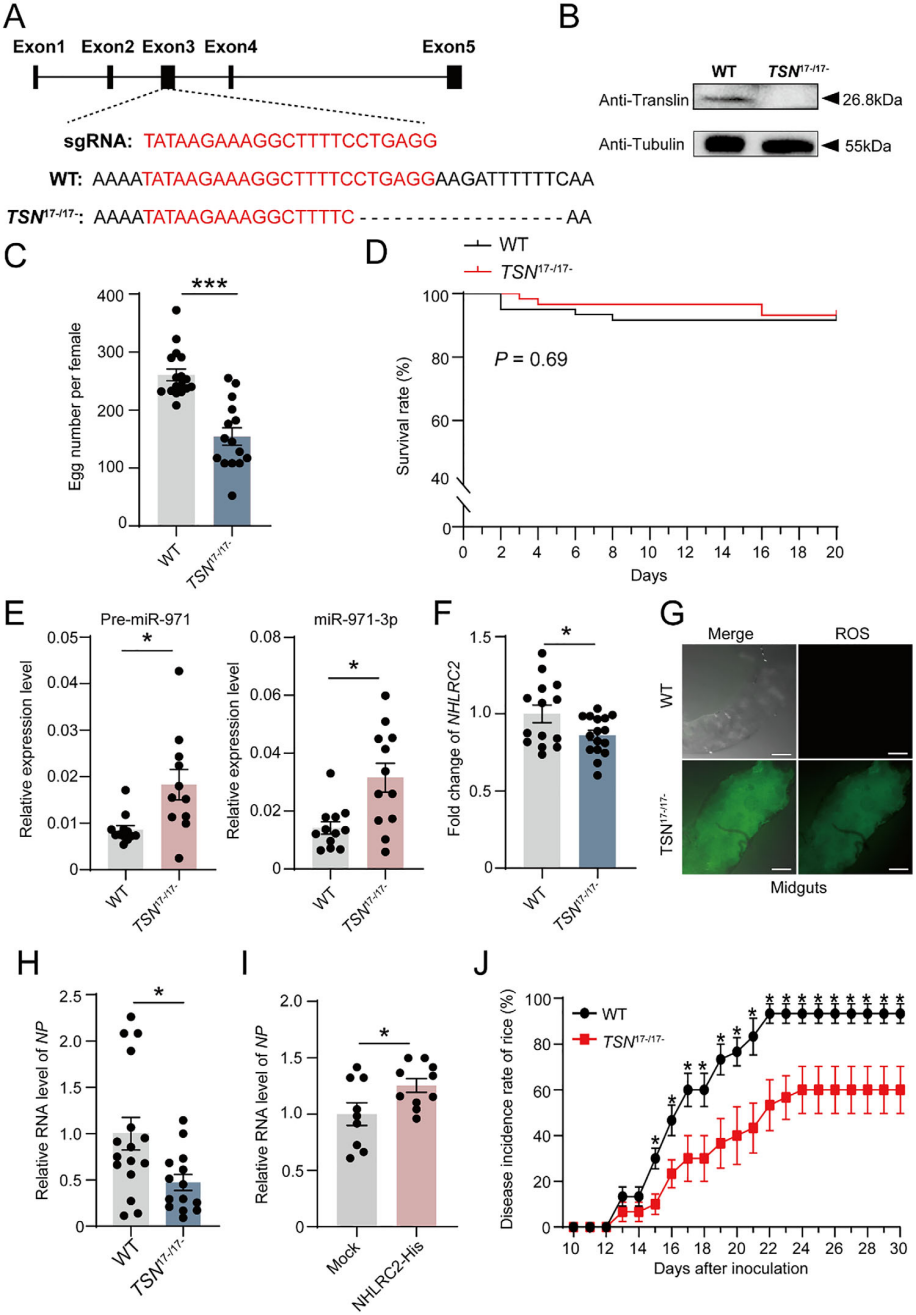
The comprehensive effects of C3PO on RSV replication and transmission. A) Germline deletion mutations in planthopper *Translin* using CRISPR‐Cas9. The target sequence is highlighted in red. The symbol “‐” represents deleted nucleotides. WT, wild‐type. *TSN* ^17‐/17‐^, homozygous mutant harboring a 17 bp deletion. B) Protein levels of Translin in the whole bodies of mutant line or WT determined by western blot using an anti‐Translin polyclonal antibody. *β*‐tubulin is used as an internal control detected by an anti‐*β*‐tubulin monoclonal antibody. C) The average number of eggs produced by a single female of mutant or WT planthoppers within 14 d. n = 15–17 for each group. D) Survival curves of mutant or WT planthoppers throughout larval stages. n = 60 for each group. E) Expression levels of pre‐miR‐971 and miR‐971‐3p relative to that of *U6* in *TSN* ^17‐/17‐^ and WT planthoppers detected by qPCR. n = 11–12 for each group. F) Fold change of *NHLRC2* transcript level in *TSN* ^17‐/17‐^ planthoppers compare to that in WT planthoppers. n = 14–16 for each group. G) ROS activities in midgut epithelial cells from WT or *TSN* ^17‐/17‐^ planthoppers. ROS activities were measured using a DCFH‐DA assay kit under a confocal microscope. Scale bars, 50 µm. “Merge” means the bright field image merging with the fluorescence signal. H) RNA level of RSV *NP* relative to that of *EF2* in mutant or WT planthoppers after fed on RSV‐infected rice for 14 d. n = 14–16 for each group. I) Fold change of RSV *NP* RNA levels in planthoppers fed NHLRC2‐His compare to that fed purified extracts from pET28a vector. n = 9–10 for each group. J) The disease incidence rates of the rice fed by RSV‐infected mutant or WT planthoppers. n = 6 for each group. For (C), (D), (E), (F), (H) (I)and (J) the values are presented as mean ± SE. Differences were statistically evaluated using Student's *t*‐test for comparison between two groups. *, *p* < 0.05. ***, *p* < 0.001.

To systemically evaluate the role of C3PO in RSV replication, we generated a *Translin* mutant strain of the small brown planthopper using the CRISPR‐Cas9 system. A single guide RNA (sgRNA) targeting the exon 3 of *Translin* (**Figure** **5**A) and Cas9 protein were microinjected into preblastoderm eggs. Five G_0_ adults were mated individually with wild‐type (WT) planthoppers to produce G_1_ eggs. After confirming the genotypes of *Translin* via Sanger sequencing, the G_1_ offsprings of the heterozygous mutant G_0_ adults were inbred for three generations. A homozygous line harboring a 17 bp deletion (*TSN* ^17‐/17‐^) in *Translin* was obtained in G_4_ (Figure 5A). Western blot assays using a homemade anti‐Translin polyclonal antibody^[^ ^28^ ^]^ demonstrated that the 17 bp deletion created frameshifts, resulting in the absence of Translin protein (Figure 5B). This mutant line exhibited a notable reduction in fecundity (Figure 5C) while maintaining a normal survival rate during the larval stages (Figure 5D).

In the *TSN* ^17‐/17‐^ mutant line, the amounts of miR‐971‐3p as well as their precursors were higher than those observed in WT (Figure 5E). The transcript level of *NHLRC2* decreased significantly in the *TSN* ^17‐/17‐^ mutants (Figure 5F), and the ROS activity in the midgut epithelial cells of the *TSN* ^17‐/17‐^ mutants increased dramatically compared to the WT planthoppers (Figure 5G). These phenotypes were consistent with those from the treatments of dsRNA or miRNA agomir.

After fed on RSV‐infected rice for 14 d, the mutants displayed a diminished RSV titer, evidenced by the 52.7% decline in the RNA level of RSV *NP* compared to WT planthoppers in the qPCR assay (Figure 5H). Upon the overexpression of *NHLRC2* in mutant planthoppers, which were administered purified NHLRC2‐His protein, a 25% increase in RSV levels was observed at 2 dpi compared to those fed with the mock samples (Figure 5I). When mutant and WT planthoppers were fed on RSV‐infected rice and subsequently transferred to healthy rice seedlings for 5 days and then removed, the rice seedlings were cultivated in a greenhouse for disease symptom observation. It turned out that the incidence of rice disease decreased from 93.3% in the WT group to 60% in the mutant group after 24 days (Figure 5J). These results showed that C3PO has a great impact on RSV replication, leading to a significant outcome in viral transmission.

### 2.6 Effects of C3PO on the Infection of Other Arboviruses in Arthropod Vectors

Considering that the C3PO complex takes part in the RNAi pathway broadly in animal kingdom,^[^ ^13^, ^14^ ^]^ we verified whether this complex, particularly Translin, affected the infection of other arboviruses in their arthropod vectors, such as sindbis virus (SINV) transmitted by mosquito *Culex quinquefasciatus* and severe fever with thrombocytopenia syndrome bunyavirus (SFTSV) vectored by tick *Haemaphysalis longicornis*. The open reading frame (ORF) of *Translin* from *C. quinquefasciatus* (*CqTranslin*) was cloned based on the sequence from GenBank (XP_001851449). We applied 5′ and 3′ rapid amplification of cDNA ends (RACE) to obtain the ORF of *Translin* from *H. longicornis* (*HlTranslin*, GenBank PQ034585). *CqTranslin* and *HlTranslin* are 711 bp and 705 bp, putatively encoding proteins of 26.8 kD and 26.6 kD.

**Figure 6:**
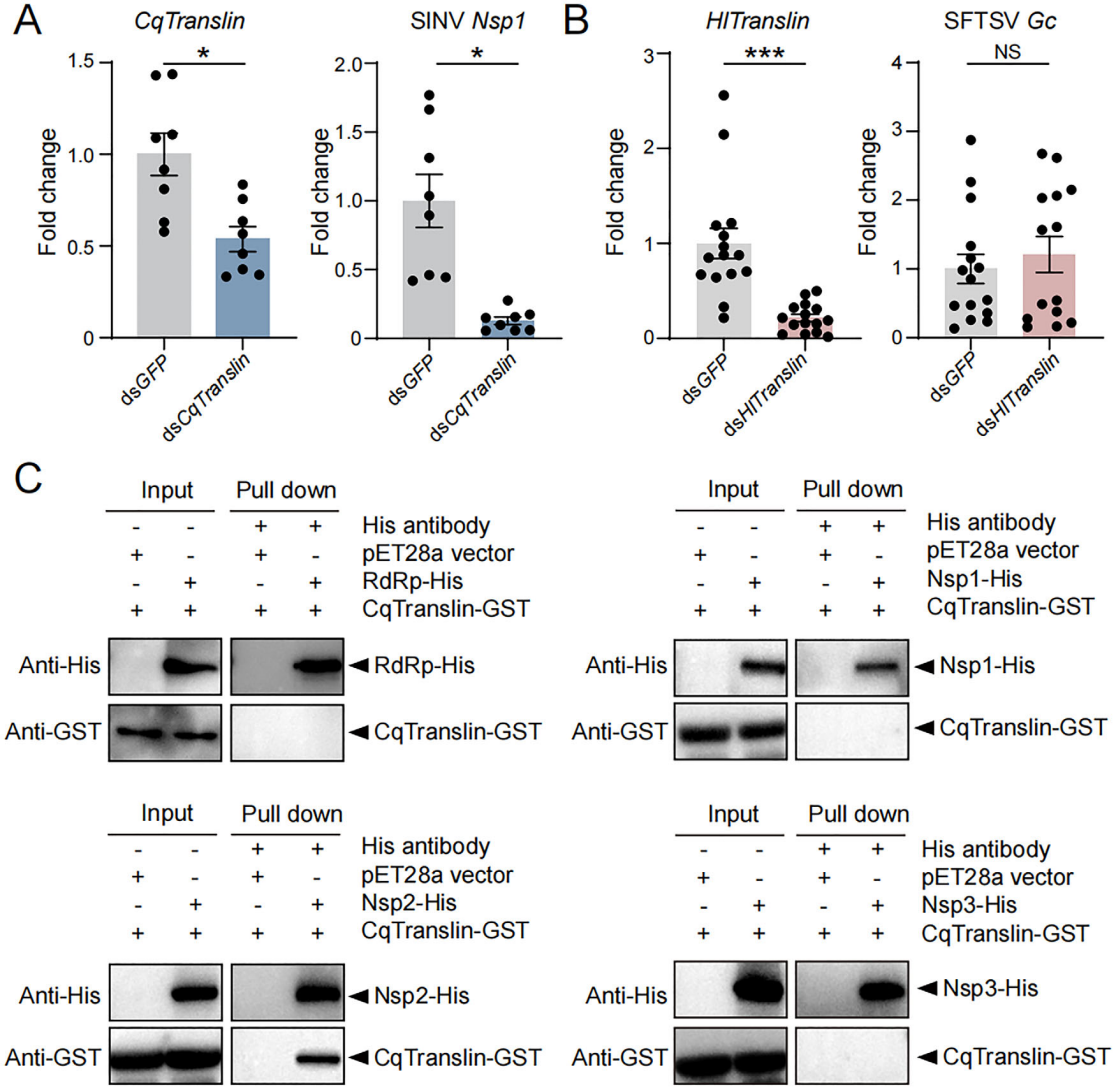
Effects of C3PO on the infection of other arboviruses in arthropod vectors. A) Fold change of *CqTranslin* transcripts and RNA levels of SINV *Nsp1* in female mosquitoes at 4 days after injection of ds*CqTranslin*‐RNA and SINV virions compared to those of injection with ds*GFP*‐RNA and virions. n = 8 for each group. B) Fold change of *Hltranslin* transcripts and RNA levels of *SFTSV Gc* in female ticks at 10 days after injection of ds*HlTranslin*‐RNA and SFTSV virions compared to those of injection with ds*GFP*‐RNA and virions. n = 14–15 for each group. For (A) and (B), the values are presented as mean ± SE. NS, no significant differences. Differences were statistically evaluated using Student's *t*‐test for comparison between two groups. *, *p* < 0.05. ***, *p* < 0.001. C) Pull down assays on the interaction between recombinant CqTranslin‐GST and SINV RdRp‐His, Nsp1‐His, Nsp2‐His, or Nsp3‐His using anti‐His monoclonal antibody. The extracts from pET28a vector serve as negative control.

The female adults of mosquitoes were injected with ds*CqTranslin*‐RNA, followed by SINV virions injection 2 days later. The silencing of *CqTranslin* expression resulted in a marked reduction of SINV as indicated by decreased RNA levels of the non‐structural protein *Nsp1* at 2 dpi (**Figure** **6**A), while the knockdown of *CqTrax* (GenBank CPIJ011091) did not exhibit a notable effect on SINV levels (Figure S6, Supporting Information). On the contrary, when the expression of *HlTranslin* was knocked down in the female adults of ticks with injection of ds*HlTranslin*‐RNA, the amount of SFTSV did not change in terms of the RNA level of *glycoprotein Gc* at 10 days after injection of SFTSV virions (Figure 6B). We further tested the interaction between *Cq*Translin and SINV proteins using pull down assays. Unlike the interaction observed between RSV RdRp and planthopper Translin, the recombinantly expressed SINV RdRp‐His (GenBank NP_740669) failed to co‐precipitate with CqTranslin‐GST (Figure 6C). Instead, Nsp2 (GenBank OR085477), one of the three non‐structural proteins of SINV, interacted with CqTranslin‐GST while Nsp1 (GenBank OR085476) and Nsp3 (GenBank NP_740672) did not (Figure 6C). These results suggest that the C3PO complex facilitates SINV replication with a potential crosstalk in its mosquito vectors, while showing no significant effect on SFTSV replication in its tick vectors.

## 3 Discussion

**Figure 7:**
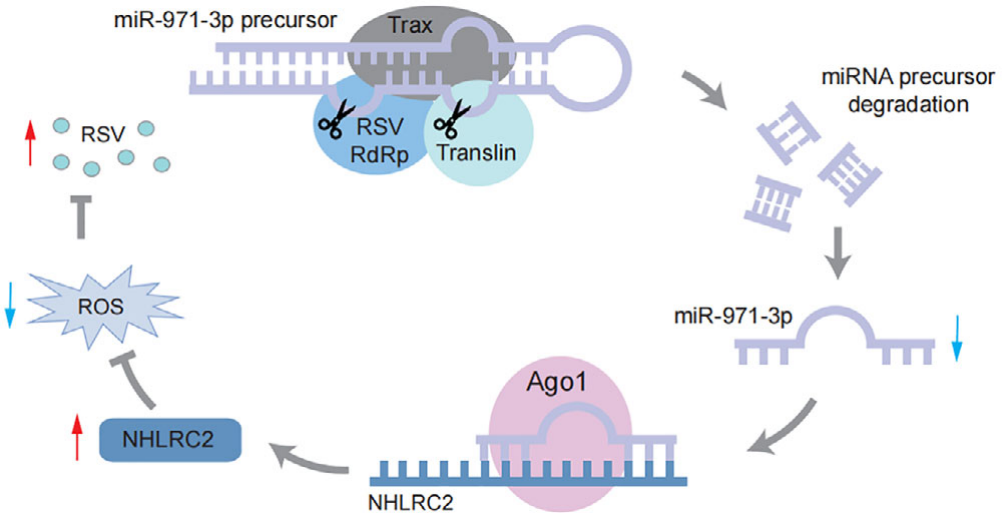
Model of arboviruses hijack C3PO‐mediated miRNA precursor degradation to promote viral infection. In small brown planthoppers, C3PO (Translin and Trax) directly degrades miR‐971‐3p precursor, leading to the reduction of mature miR‐971‐3p. With RSV acquisition, the RdRp of the virus binds C3PO and amplifies the degradation activity of C3PO toward miR‐971‐3p precursor. The decrease of miR‐971‐3p activates the expression of target gene *NHLRC2*, which abolishes ROS to facilitate RSV replication.

In this study, we elucidated an important role of the C3PO complex in the infection dynamics of arboviruses within their insect vectors. In small brown planthoppers, C3PO was shown to directly degrade miRNA precursors, especially miR‐971‐3p, resulting in diminished levels of mature miR‐971‐3p. During the acquisition of RSV, the viral RdRp interacted with C3PO, enhancing its degradation activity against miR‐971‐3p precursors. The reduction in miR‐971‐3p subsequently activated the expression of the target gene *NHLRC2*, which scavenged ROS to promote RSV replication (**Figure** **7**). This research broadens our understanding of the roles of this ubiquitous complex in various animal species and the immune interplay between arboviruses and their transmission vectors.

C3PO is implicated in numerous biological processes in animals. Translin and Trax are conserved across a range of organisms, including mammals, insects, and plants.^[^ ^34^, ^35^, ^36^ ^]^ In mammals, Translin participates in chromosomal translocation, telomere maintenance, cell proliferation and differentiation, and male fertility.^[^ ^37^, ^38^, ^39^, ^40^, ^41^ ^]^ Trax plays a role in regulating axonal regeneration, promoting cell proliferation, blocking *PLCβ1* activity and optimizing DNA repair.^[^ ^42^, ^43^, ^44^, ^45^ ^]^ In human and rat neuronal cell lines, C3PO stabilizes the cytoplasmic localization of *PLCβ*, resulting in a reduction of Ca^2+^ release in response to *Gα_q_/PLCβ* stimulation.^[^ ^46^ ^]^ Furthermore, C3PO functions as a nucleic acid endonuclease to affect miRNA and siRNA processing.^[^ ^13^, ^14^ ^]^ In mice, activation of C3PO causes a decline in miR‐181b levels, leading to aortic stiffening via the promotion of the TGF‐β signaling pathway.^[^ ^47^ ^]^ Deletion of C3PO increases the accumulation of miR‐bantam in *Drosophila*.^[^ ^48^ ^]^ Our findings demonstrated that C3PO benefits for arboviral infection in insect vectors by directly cleaving miRNA precursors, allowing arboviruses to exploit and cooperate with this complex to enhance their replication. Simultaneously, a paradox emerges. The depletion of *Translin* led to a reduction in RSV levels, whereas the knockdown of *Trax* exhibited no significant impact. This discrepancy may stem from the differential influence of the two proteins on each other. For mammal and *Drosophila* C3PO complex, the absence of Translin results in a complete or nearly complete depletion of Trax protein,^[^ ^17^, ^35^, ^38^, ^49^ ^]^ while the mRNA expression of *Trax* remains unaltered.^[^ ^49^ ^]^ Conversely, the deletion of Trax does not induce a comparable decline in Translin protein.^[^ ^17^ ^]^ This implies that Translin plays a leading role for the functionality of the C3PO complex. In our study, temporary interference of *Translin* expression in planthoppers could affect the protein levels of both Translin and Trax, while temporary interference of *Trax* did not yield significant reductions in either protein. We will verify this hypothesis in future upon the availability of an antibody specific to Trax.

C3PO inhibits miRNA production with certain substrate specificity. It has been reported that C3PO targets and cleaves the miRNA precursors characterized by mismatch structures in the stem region.^[^ ^14^ ^]^ We found that four miRNAs were upregulated upon partial suppression of C3PO in small brown planthoppers, each possessing mismatch structures in their stem regions. Similarly, miRNA profiling of mouse aortic tissues showed that inhibition of C3PO leads to an increase in only a limited subset of miRNAs.^[^ ^20^ ^]^ Additionally, there are two alternative miRNA degradation pathways in mammals. The monocyte chemoattractant protein 1‐induced protein 1 (MCPIP1) suppresses the biosynthesis of a broad range of miRNA species via preferential cleavage of the unpaired regions around the terminal loops of miRNA precursors.^[^ ^50^ ^]^ The RNA‐binding protein Lin‐28 specifically recognizes and binds to a GGAG motif present in the terminal loop of precursors of let‐7 family members, recruiting TUT4/Zcchc11 to uridylate the 3′ terminal of these miRNA precursors, which are subsequently degraded by Dis312.^[^ ^51^ ^]^ These three miRNA degradation pathways do not compromise the fundamental molecular components of the miRNA pathway and only play a regulatory role in miRNA biosynthesis. Based on our findings and previous references, we propose that the three miRNA degradation pathways are virus‐regulating hotspots. RSV accelerates C3PO's cleavage to miR‐971‐3p via RdRp for the benefit of viral replication in vector insects. C3PO also facilitates SINV infection in vector *Culex* mosquitoes and viral Nsp2 is capable of binding C3PO, though the implications of this interaction remain unclear. Hepatitis B virus (HBV) induces Lin‐28 homolog to downregulate let‐7 expression in HepG2 hepatoma cells, potentially hindering HBV replication.^[^ ^52^, ^53^ ^]^ MCPIP1 inhibits Kaposi's sarcoma‐associated herpesvirus (KSHV) infection by directly degrading viral miRNA precursors.^[^ ^54^ ^]^ However, *MCPIP1* expression is repressed upon KSHV infection.^[^ ^55^ ^]^

The planthopper NHLRC2 exhibits thioredoxin reductase activity, enabling the reduction of ROS. Its N‐terminal thioredoxin domain encompasses an oxidoreductase motif CXXC (CINC). The recombinantly expressed NHLRC2 shows a thioredoxin reductase activity, corresponding to its capability of eliminating H_2_O_2_ and other ROS. In contrast, human NHLRC2 does not exhibit thioredoxin reductase activity although it contains an N‐terminal thioredoxin‐like domain, suggesting that human NHLRC2 might not be involved in thiol‐disulfide exchange.^[^ ^56^ ^]^ In mammals, the functions of NHLRC2 are associated with cell differentiation, survival, and embryo development. Mutated *NHLRC2* enhances the differentiation of fibroblasts to myofibroblasts, leading to increased fibrosis in human tissues.^[^ ^57^ ^]^ The high expression of *NHLRC2* reduces disease‐specific survival, overall survival, and high mitotic activity in lung adenocarcinoma.^[^ ^58^ ^]^ A deficiency in *NHLRC2* results in failure of gastrulation and amniotic folding in mice.^[^ ^59^ ^]^ Furthermore, NHLRC2 regulates phagocytosis in macrophages by influencing actin dynamics.^[^ ^60^ ^]^ Mutated *NHLRC2* exhibits high resistance to *Salmonella* infections.^[^ ^56^ ^]^ However, the involvement of NHLRC2 in viral infections remains to be elucidated in mammals. Due to its thioredoxin reductase activity, planthopper NHLRC2 functions as an antioxidant to regulate ROS levels in midgut epithelial cells. In addition to *NHLRC2*, previous studies showed that RSV infection induced the expression of multiple antioxidant genes (such as *peroxidorexin*, *cytochrome P450*, and *cathepsin B*) in planthoppers.^[^ ^61^ ^]^ However, the ROS levels in viruliferous planthoppers were still significantly higher than in nonviruliferous planthoppers. Perhaps this level of ROS perfectly maintains the balance between viral titer and insect immunity. In the absence of activation of these antioxidant genes, increased ROS levels would significantly hinder RSV replication. Interestingly, a moderate ROS concentration can occasionally facilitate viral infection. For example, ROS‐induced autophagy slightly enhances the replication of *Bombyx mori* nucleopolyhedrovirus (BmNPV) during the early infection stage, whereas excessive ROS accumulation triggers apoptosis and suppresses BmNPV replication in later stages.^[^ ^62^ ^]^ H_2_O_2_ activates turnip mosaic virus (TuMV) transmission through the induction of intermolecular cysteine bonds between viral helper component protease (HC‐Pro) molecules and the formation of viral transmission complex which is composed of TuMV particles and HC‐Pro.^[^ ^63^ ^]^ Elevated ROS levels increases susceptibility of the brain to SINV infection by disrupting the septate junctions and compromising blood‐brain barrier integrity.^[^ ^64^ ^]^

In summary, we demonstrate that C3PO‐mediated miRNA degradation pathway in insect vectors plays a critical role in arbovirus infection, which arboviruses also exploit for their advantage. Therefore, targeting C3PO or *NHLRC2* would be considered as a promising strategy in controlling arbovirus infection.

## 4 Experimental Section

### Insect Strains and Virus Preparation

The small brown planthopper strains were derived from a field population collected from Nanjing, Jiangsu Province, China. Nonviruliferous and viruliferous planthoppers were cultured on *Oryza sativa* Wuyujing in glass incubators at 25 ± 1 °C with a photoperiod 16 h of light per day. To achieve an RSV‐carrying rate of at least 80%, a homemade anti‐NP monoclonal antibody was utilized to screen for virus‐carrying planthoppers every 3 months through a dot enzyme‐linked immunosorbent assay (dot‐ELISA) as described by Zhao et al.^[^ ^65^ ^]^

The *C. quinquefasciatus* strain was maintained in an incubator at 28 ± 1 °C with a photoperiod (16 h light / 8 h darkness). SINV virus strain (YN87448) was cultured in *Aedes albopictus* C6/36 cells (RRID: CVCL_Z230) using 1640 medium (C11875500BT, Gibco, Thermo Fisher Scientific, Waltham, MA, USA) supplemented with 8% fetal bovine serum (FBS) at 28 °C with 5% CO_2_. The *H. longicornis* strain was collected in Haikou, Hainan Province, China, and maintained at 25 ± 1 °C. SFTSV Wuhan strain was gifted by Wuhan Institute of Virology, Chinese Academy of Sciences, and was inoculated in Vero cells (RRID: CVCL_0059) with Dulbecco's modified Eagle's medium (DMEM) supplemented with 10% FBS at 37 °C with 5% CO_2_.^[^ ^66^ ^]^

### Injection of RSV Crude Preparations

Sixty viruliferous adult planthoppers were homogenized in 100 µL of 10 mM phosphate‐buffered saline (PBS, pH 7.4). The mixture was centrifuged at 13 000 rpm for 15 min at 4 °C, and the supernatant was retained. This process was repeated five to six times, and the supernatant from the final centrifugation was used as the crude RSV preparation. A 23 nL of RSV crude preparation was injected into nonviruliferous third‐instar nymphs through a glass needle using a Nanoliter 2000 microinjector (World Precision Instruments). Then, the insects were respectively collected at 8 dpi. seven to eight biological replicates and five nymphs or ten midguts per biological replicate were prepared for qPCR assay.

### DsRNA Synthesis and Injection

A 498 bp of dsRNA for planthopper *Translin*, 485 bp of dsRNA for planthopper *Trax*,437 bp of dsRNA for planthopper *NHLRC2*, 202 bp of dsRNA for *CqTranslin*, 319 bp of dsRNA for *CqTrax*, 307 bp of dsRNA for *Hltranslin*, and 420 bp of dsRNA for *GFP* were synthesized using the T7 RiboMAX Express RNAi System (Promega, Madison, WI, USA) following the manufacturer's protocol. Primers were listed in Table S4, Supporting Information. A volume of 23 nL of dsRNA at 6 µg µL^−1^ was injected into third‐instar viruliferous planthoppers using a Nanoliter 2000 microinjector (World Precision Instruments). These planthoppers were collected at 4 dpi or 6 dpi for RNA extraction, qPCR, and western blot assays. Six to twelve biological replicates and five nymphs, ten midguts or ten salivary glands per replicate were prepared.

### Injection of dsRNAs and SINV or SFTSV Virions in Mosquitoes or Ticks

Mosquito female adults aged 6 to 8 days post eclosion were first injected with 150 nL of ds*CqTranslin*‐RNA, ds*CqTrax*‐RNA or ds*GFP*‐RNA at 10 µg µL^−1^, and after 2 d, they were injected with 150 nL of SINV virions at 30 000 pfu ml^−1^ using a Nanoliter 2000 microinjector (World Precision Instruments). These mosquitoes were collected at 2 dpi for the quantification of transcript level of *CqTranslin*, *CqTrax* and RNA level of SINV *Nsp1* using qPCR. Eight to eleven biological replicates and one adult per replicate were prepared. Non‐blood fed female adult ticks aged 1.5 months post‐eclosion were delivered with 0.5 µL of ds*HlTranslin*‐RNA or ds*GFP*‐RNA at 2 µg µL^−1^. 6 days later, they were injected with 0.5 µL of SFTSV virions at 7.25 × 10^3^ FFU using Nanofil (World Precision Instruments). These ticks were collected at 10 dpi for the quantification of the transcript level of *HlTranslin* and the RNA level of SFTSV *Gc* by qPCR. Fourteen to Fifteen biological replicates and one adult per replicate were prepared.

### RNA Extraction And Reverse Transcription

Total RNA was extracted from whole bodies of 5 planthoppers, 10 midguts, 10 salivary glands, 1 adult tick, or 1 adult mosquito using TRIzol Reagent (Invitrogen) according to the manufacturer's instructions. One microgram of total RNA was utilized for reverse transcription with MLV reverse transcriptase and random primers (Promega) for normal cDNA synthesis or with miRNA cDNA First Strand Synthesis Kit (Tiangen, Beijing, China) for miRNA cDNA synthesis following the manufacturer's instructions.

### Small RNA‐Seq Analysis

Small RNA libraries were constructed using total RNAs isolated from third‐instar viruliferous planthoppers at 4 and 6 days post injection of ds*Translin*‐RNA or ds*GFP*‐RNA and sequenced in Beijing Genome Institute (BGI, Shenzhen, China). Two biological replicates for each group were sequenced. Small RNA libraries was constructed on the BGISEQ‐500 platform to generate 50 bp‐end reads in Beijing Genome Institute (BGI, Shenzhen, China). After data filtering, clean reads were mapped to *L. striatellus* genome and databases including miRBase (http://microrna.sanger.ac.uk), Rfam (http://rfam.xfam.org), siRNA, piRNA (http://ento.njau.edu.cn/Piano.html), and snoRNA (https://www‐snorna.biotoul.fr/). Small RNAs with a length distribution between 18 nt and 30 nt were selected for subsequent analysis. MiRDeep2 was used to predict novel miRNAs.^[^ ^67^ ^]^ The expression levels of small RNAs were quantified based on unique molecular identifier (UMI) species numbers. Differentially expressed miRNAs were screened by Deseq2.^[^ ^68^ ^]^ The miRNA expression level was normalized by comparing the sequences in each sample to the miRNA library created in this study, using transcripts per million (TPM) as the assessment measure. TPM was calculated as (numbers of each miRNA matched to total reads)/(number total reads) × 10^6^. The significance difference was judged by the threshold of adjusted *p* value < 0.05 and twofold change.

### Recombinant Protein Expression and Purification

The ORFs of planthopper *Translin*, *Trax*, *NHLRC2*, RSV *RdRp2*, and SINV *Nsp1*, *Nsp2*, *Nsp3*, and *RdRp* were inserted into the pET28a vector to generate recombinant plasmids with a His‐tag. The *CqTranslin* were introduced into the pGEX3X vector to generate recombinant plasmids with a GST‐tag. Primers were listed in Table S4, Supporting Information. The recombinant plasmids were transformed into *Escherichia coli* strain BL21 for protein expression, as described by Zhu et al.^[^ ^69^ ^]^ After 16 h of induction with 0.5 mM isopropyl β‐D‐thiogalactoside (IPTG) at 16 °C, cells were sonicated for 30 min at 4 °C. The supernatant was retained for protein purification. Translin‐His, Trax‐His, RdRp2‐His, and NHLRC2‐His were purified using Ni Sepharose (GE Healthcare, Buckinghamshire, UK) following the manufacturer's instructions and dissolved in 10 mM PBS (pH 7.4) after filtration with a 10‐kDa cutoff Amicon Ultra Centrifugal Filter (Millipore). The purified Translin‐His, Trax‐His, RdRp2‐His was subsequently used in pre‐miRNA degradation and the purified NHLRC2‐His was used in TrxR enzyme activity assays.

### Pull Down Assay

The in vitro pull down assays were performed as described by Zhu et al.^[^ ^69^ ^]^ Five micrograms of anti‐His monoclonal antibody (CWBiotech, Beijing, China) was incubated with 50 µL of Dynabeads Protein G (Novex, Thermo Fisher Scientific) for 30 min at 4 °C. A 400 µL 1:1 mixture of two recombinantly expressed proteins were added and incubated at 4 °C for 2 h. The extracts from pET28a vector was used as negative controls. The pulled down proteins were detected through western blot assay using anti‐His monoclonal antibody (CWBiotech) and anti‐GST monoclonal antibody (EASYBIO, Beijing, China).

### Extraction of Cytoplasmic and Nuclear Fractions

Cytoplasmic and nuclear fractions were extracted from viruliferous nymphs at 4 days after injection with ds*Translin*/ds*GFP* using a nuclear and cytoplasmic extraction kit (BestBio, Shanghai, China). A total of 20 nymphs were homogenized in 200 µL of PBS (pH7.4) using a TGrinder high‐speed tissue grinder (Tiangen Biotech, Beijing, China). Following a 20‐min incubation on ice, the homogenates were centrifuged at 500 × g for 5 min at 4 °C and the supernatant was discarded. 200 µL of extract buffer A and 0.8 µL of protease inhibitor Cocktail were added to the cell precipitation. After a 30‐min incubation at 4 °C, the mixture was centrifuged at 12 000 × g for 10 min at 4 °C. The supernatant was retained as a cytoplasmic protein. Subsequently, 200 µL of extract buffer B and 0.8 µL of protease inhibitor cocktail was added to the precipitation. After incubating at 4 °C for 30 min, the samples were centrifuged at 12 000 × g for 10 min at 4 °C. The supernatant was retained as a nuclear protein. Both cytoplasmic and nuclear extracts were then subjected to Western blot analysis and RNA extraction. For the detection of nuclear and cytoplasmic proteins, histone H3 and GAPDH were used as markers, respectively, with monoclonal anti‐H3 antibody (EASYBIO, Beijing, China) and polyclonal anti‐GAPDH antibody (Abcam, Cambridge, UK).

### Pre‐miRNA Degradation Assay

The 5′biotin‐labeled miRNA precursors and their corresponding mutants were synthesized in Beijing Genome Institute (BGI, Shenzhen, China). For the degradation of pre‐miRNAs by C3PO, the 50‐µL reaction was comprised of 10 µL of 5× buffer A (500 mM KCl, 100 mM Tris, and 15 mM MgCl_2_), 500 ng of pre‐miRNA and 0 to 10 nM C3PO. For the degradation of pre‐miRNAs by RSV RdRp2 with C3PO or without C3PO, the 50‐µL reaction was comprised of 10 µL of 5× buffer A plus 5 mM MnCl_2_, 500 ng of pre‐miRNA, 0 to 10 nM RSV RdRp2 and 5nM C3PO or without C3PO. For the degradation of pre‐miRNAs by WT‐RdRp2 or mutant RdRp2, the 50‐µL reaction was comprised of 10 µL of 5× buffer A plus 5 mM MnCl_2_, 250 ng of pre‐miRNA and 8 nM WT‐RdRp2 or mutant RdRp2. The *RdRp2* mutant was constructed using the KOD‐Plus mutagenesis kit (Toyobo, Japan). The purified extracts from pET28a vector were employed as negative control. After incubated at 37 °C for 30 min, the reaction product was extracted with phenol chloroform and precipitated with 95% ethanol. Products were electrophoresed on a 16% polyacrylamide gel containing 7 M urea and transferred to a Biodyne B Nylon Membrane (Thermo Fisher Scientific). The biotin signal was detected using HRP‐labeled streptavidin and the SuperSignal West Femto Chemiluminescence ECL Kit (Thermo Fisher Scientific).

### Injection of miR‐971‐3p Agomir and Antagomir

A 23 nL of miR‐971‐3p/NC agomir at 250 µM or miR‐971‐3p/NC antagomir at 500 µM (GenePharma, Shanghai, China) was delivered into third‐instar viruliferous or nonviruliferous planthopper nymphs by microinjection using a Nanoliter 2000 microinjector (World Precision Instruments). At 6 dpi, five planthoppers each replicate and six to eleven replicates were collected for qPCR. Western blot assay was performed for measuring the protein level of RSV NP using a homemade anti‐NP monoclonal antibody. Anti‐*β*‐tubulin antibody (EASYBIO) was used for internal control. The grayscales of NP and tubulin bands were quantified with ImageJ software and the relative grayscale of NP to that of tubulin was compared between groups. In a rescue experiment, third‐instar viruliferous planthoppers were co‐injected with ds*NHLRC2*‐RNA and miR‐971‐3p antagomir. At 6 dpi, the expression levels of *NHLRC2* transcripts, RSV *NP* RNA and miR‐971‐3p in the whole bodies were quantified by qPCR. Five planthoppers each replicate and six to eight replicates were collected.

### MiR‐971‐3p Target Gene Prediction

MiRanda and RNAhybrid algorithms were employed to predict the potential binding sites of miR‐971‐3p in UTRs and CDS of planthopper genes. The minimum free energy (MFE) of the RNA duplex was set with a cutoff value of −20 kcal mol^−1^ in both algorithms.

### Dual Luciferase Assay

≈300 bp fragment around the target site of *NHLRC2* was inserted into the Psi‐CHECK2 vector (Promega; C8021) between the restriction sites *Not*I and *Xho*I to generate recombinant plasmids. Mutations in the target site of *NHLRC2* were introduced using the KOD‐Plus mutagenesis kit. MiR‐971‐3p mimic and NC mimic (5′‐UUCUCCGAACGUGUCACGUTT‐3′) were synthesized in GenePharma company. *Drosophila* S2 cells (RRID: CVCL_Z232) were co‐transfected with 1µg of plasmid and 50 nM miR‐971‐3p/NC mimic using Lipofectamine 3000 system (Invitrogen). After 24 h of transfection at 28 °C, cells were collected for the measurement of firefly and Renilla luciferase activities using the Dual‐Glo luciferase assay system (Promega). Eight replicates were prepared for each group. The relative activity of Rluc normalized to Fluc activity is presented as the mean ± SE. The primers used in this experiment are listed in Table S4, Supporting Information.

### RIP‐qPCR assay

A 23 nL of miR‐971‐3p/NC agomir at 250 µM was injected into viruliferous third‐instar planthopper nymphs, and after 4 days the nymphs were collected for RIP assay using an RNA immunoprecipitation kit (BersionBio, Guangzhou, China) as previously described.^[^ ^70^ ^]^ The homogenized extracts from 35 planthoppers were incubated with 2 µg of a homemade anti‐Ago1 antibody or IgG antibody at 4 °C overnight. Ten percent of the lysate supernatant was served as the “Input” sample for reference. Total RNA was extracted from input samples or Ago1/IgG‐pull down samples using TRIzol Reagent (Invitrogen), followed by reverse transcription and qPCR for the detection of miR‐971‐3p and *NHLRC2*. Six replicates were prepared. The RNA level of each target RNA relative to that in the IgG control sample is reported as the mean ± SE.

### Thioredoxin Reductase Assay

The 300 µg purified NHLRC2‐His protein was incubated with NADPH and DTNB (5,5′‐dithiobis (2‐nitrobenzoic) acid) at 37 °C for 5 min according to the instructions of the thioredoxin reductase assay kit (Ak129, Biosynthesis biotechnology, Beijing, China). The thioredoxin reductase activity was evaluated by measuring the OD 412 nm corresponding to TNB formation. Equal amount of bovine serum albumin (BSA) was employed as a negative control. Six replicates were prepared.

### ROS and H_2_O_2_ Content Measurement

The ROS levels were determined using a general oxidative stress indicator DCFH‐DA (2′,7′‐dichlorodihydrofluorescein diacetate, Sigma‐Aldrich, Alexandria, VA) according to the manufacturer's instructions. The midguts and salivary glands were incubated with 10 µM DCFH‐DA in the dark at room temperature for 40 min, and then washed three times with 10 mM PBS (pH 7.4). The excitation wavelength for green fluorescence (DCF) was set at 488 nm, with the emission wavelength for detection set at 525 nm. Bright field and fluorescence images were captured under a Zeiss LSM 710 confocal microscope (Carl Zeiss AG). For the H_2_O_2_ sensitivity assay, 0.03 g of tissue from nonviruliferous planthoppers, viruliferous planthoppers, or nymphs at 6 days after injection with ds*NHLRC2*/ds*GFP* was homogenized with 100 µL of acetone. After centrifugation, the supernatant was collected to measure H_2_O_2_ activities using a Hydrogen Peroxide (H_2_O_2_) Content Assay Kit (Sangon, Shanghai, China) according to the manufacturer's instructions. Seven to eleven biological replicates and 20 to 30 planthoppers per replicate were prepared. H_2_O_2_ activity was calculated as the means ± SE.

### ROS Inhibitor Treatment

Aliquots of 23 nL of NAC at 10 mM was injected into nonviruliferous third‐instar nymphs, and then these nymphs were fed on RSV‐infected rice seedlings for 6 days. Eight biological replicates with five planthoppers per replicate were collected for the determination of the RNA level of RSV *NP* and midguts were used for ROS measurement.

### 5′ RACE and 3′RACE

SMARTer RACE 5′/3′ kit (TaKaRa) was applied to obtain the full‐length ORF of *HlTranslin* according to the manufacturer's instructions. PCR reaction was performed with 25 µL of 2 × LA taq polymerase (Takara), 2.5 µL of 5′ or 3′ first strand cDNA, 1 µL of 5′ or 3′ specific primers of *HlTranslin*, and 5 µL of 10×UPM. The cycling conditions were as follows: 95 °C for 5 min, 35 cycles at 95 °C for 30 s, 68 °C for 30 s, 72 °C for 3 min, eventually 72 °C incubation for 10 min. The PCR product was purified by Wizard SV gel and PCR cleanup system (Promega) and inserted into the pGEM‐Teasy cloning vector (Promega) for sequencing. The primers used in this experiment are listed in Table S4, Supporting Information.

### Knockout of *Translin* in *L. striatellus* using CRISPR‐Cas9 Technique

We applied the CasOT^[^ ^71^ ^]^ to search the sgRNAs and their potential off‐target sites in the genome of small brown planthopper using *Translin* sequence. The search criteria for sgRNAs were a length of 20‐nt, and an NGG motif at the 3′‐end (PAM, protospacer‐adjacent motif). The maximum number of mismatches allowed in the seed and non‐seed regions were 0 and 4, respectively. The sgRNA with the least off‐target potential was predicted to target the exon 3 of *Translin* and synthesized using the GeneArt Precision gRNA Synthesis Kit (Invitrogen, Carlsbad, CA, USA) according to manufacturer's instructions. Eggs were collected from rice stem sheathes within 2 h after oviposition and lined upon an AGAR plate (2% agar containing 0.005‰ methylene blue) in a 9 cm diameter petri dish. The Cas9‐sgRNA mixture containing 100 ng/µL GeneArt Platinum Cas9 protein (Invitrogen) and 100 ng ul^−1^ sgRNA was injected into the middle of individual eggs as described previously.^[^ ^72^ ^]^ Screening mutants and construction of homozygous mutant lines followed the methods described previously.^[^ ^72^ ^]^ A homozygous *Translin* mutant line was successfully established after confirmation with Sanger sequencing and western blot assays using a homemade anti‐Translin polyclonal antibody.

### Fecundity and Survival Rate of Planthoppers

Single mated females were raised on five rice seedings and eggs were collected within 14 d. The average number of eggs from 15 *TSN* ^17‐/17‐^ mutants and 17 WT planthoppers were reported as mean ± SE. For survival rate analysis, 60 first‐instar nymphs of *TSN* ^17‐/17‐^ mutants or WT planthoppers were individually raised on three rice seedlings. The survival status of each insect was recorded daily for 20 d. Survival curves were evaluated using the Kaplan Meier method, and compared statistically using the Mantel‐Cox Log‐rank test.

### RSV Titer Measurement in Planthoppers and Rice Disease Incidence Assay

After nonviruliferous third‐instar nymphs of the *TSN* ^17‐/17‐^ mutants or WT planthoppers fed on RSV‐infected rice for 14 d, the RNA level of RSV *NP* was measured using qPCR. Five insects each replicate and fourteen to sixteen biological replicates were prepared in each group. Additionally, the recombinant NHLRC2‐His protein was administered to *TSN* ^17‐/17‐^ mutants that had been inoculated with RSV‐infected rice. 2 days post‐administration, the impact of NHLRC2 overexpression on RSV levels were assessed by quantifying the RNA levels of RSV *NP* using qPCR. The control group was fed purified extracts from pET28a vector. Five planthoppers each replicate and nine to ten biological replicates were prepared for qPCR assay. Nonviruliferous third‐instar nymphs of the *TSN* ^17‐/17‐^ mutants or WT planthoppers were fed on RSV‐infected rice for 14 days and then transferred to healthy rice seedlings for 5 d. After removal of these planthoppers from rice, the rice seedlings were cultivated in a greenhouse at 30 °C for disease symptom observation. Five rice plants each replicate and six replicates were used to calculate rice disease incidence rate.

### qPCR

The expression levels of insect genes and viral genes were detected by LightCycler 480 SYBR Green I Master (Roche, Basel, Switzerland), and the expression levels of miRNA were detected by miRcute miRNA qPCR detection kit (Tiangen). *EF2* was used as the internal reference gene of planthopper mRNAs and RSV *NP*. *β‐actin* was used as the internal reference gene of mosquito mRNAs and SINV *Nsp1*. *ELFA* was used as the internal reference gene of tick mRNAs and SFTSV *Gc*. *U6* was used as the internal reference gene of miRNAs. The qPCR reaction system was a 10‐µL volume, which included 5 µL of 2 × SYBR Green I Master Mix (Roche) or 2 × miRcute miRNA premix (Tiangen), 2 µL of cDNA template, and 0.25 µL of primers at 10 µM. qPCR was run on a LightCycler 480 II (Roche) following the manufacturer's instructions. The transcript level of each gene relative to that of the internal reference gene was calculated by 2^–ΔCt^ method.^[^ ^73^ ^]^

### Statistics

Data were analyzed using GraphPad Prism 8.3.0 and presented mean ± standard error of the mean (SEM). To evaluate the normality of the data, a Kolmogorov–Smirnov test was conducted. For data exhibiting a normal distribution, differences were statistically evaluated Student's *t*‐test for comparison between two groups and one‐way ANOVA followed by Tukey's test for multiple comparisons. Survival curves were evaluated using the Kaplan Meier method, and compared statistically using the Mantel‐Cox Log‐rank test. The statistical test method for each figure was indicated in the figure legend. Statistical significance was defined as *p < 0.05, **p < 0.01, ***p < 0.001.

## Conflict of Interest

The authors declare no conflict of interest.

## Author Contributions

Conceptualization: F.C. Investigation: Y.X., T.G., Q.X., C.C., Q.W., and H.L. Drawing: L.L. Writing original draft: Y.X. and H.L. Finalizing the draft: H.L. and F.C.

## Supporting information



Supporting Information

## Data Availability

The data that support the findings of this study are available in the supplementary material of this article.
