# Supplementary material for: RNAi Regulator C3PO Promotes Arbovirus Infection in Insect Vectors
Source: Adv Sci (Weinh). 2025 Oct 31;13(4):e15869. doi: 10.1002/advs.202515869 (PMC12822448; doi:10.1002/advs.202515869)
Supplement: Supplementary file 1 — Supporting Information [file ADVS-13-e15869-s001.docx]

**Supplemental information**

**RNAi regulator C3PO promotes arbovirus infection in insect vectors**

Yan Xiao^1,3^, Tianyu Guan^1,3^, Qianfeng Xia^2^, Chen Chen^2^, Qian Wang^1,3^, Lan Luo^1^, Hong Lu^1*^, Feng Cui^1,3*^

This PDF includes Figure S1 to S6, Table S2 and Table S4.


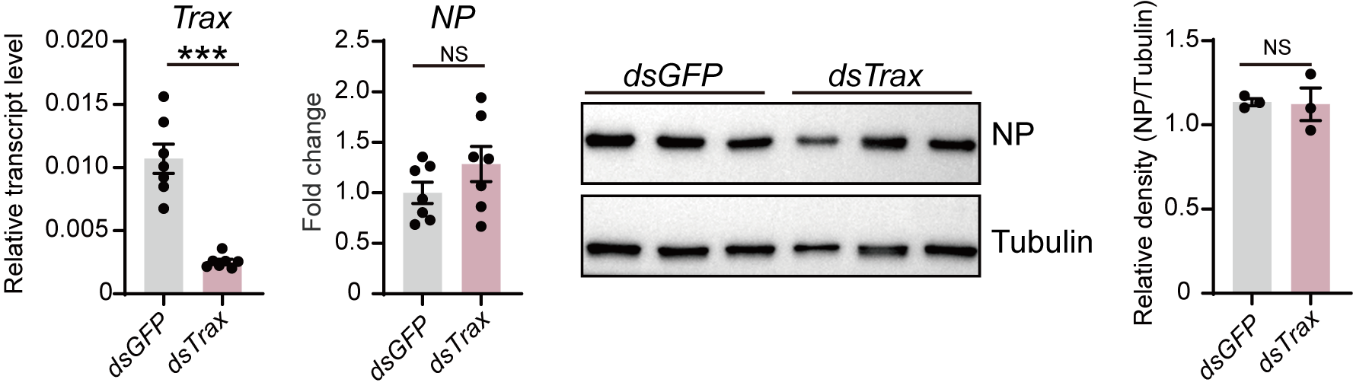


**Figure S1.** The transcript levels of *Trax* relative to that of *EF2* and fold change of RSV *NP* of viruliferous planthoppers at 6 d after injection of ds*Trax*-RNA compared to those of injection with ds*GFP*-RNA. Protein levels of RSV NP in the samples analyzed with western blot using an anti-NP monoclonal antibody. *β*-tubulin is measured as internal control using an anti-*β*-tubulin monoclonal antibody. n = 3-7 for each group. The grayscale of NP relative to that of *β*-tubulin is compared between groups. The values are presented as mean ± SE. Differences were statistically evaluated Student’s *t*-test for comparison between two groups. NS, no significant differences. ***, *p* < 0.001.


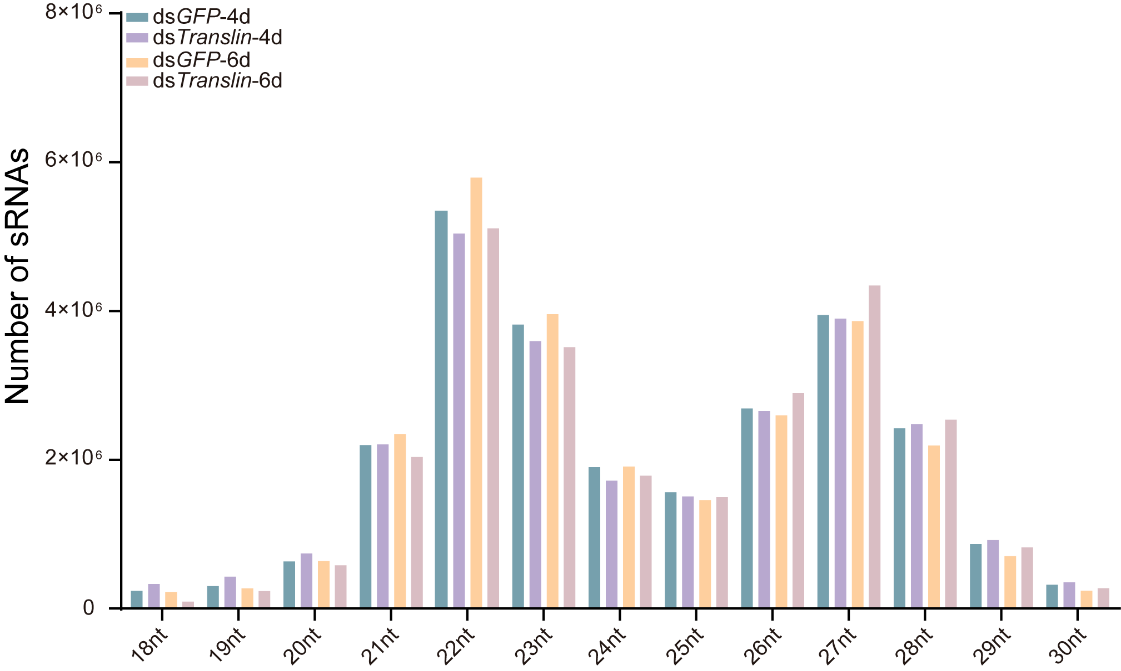


**Figure S2.** Length distribution of sRNAs in viruliferous planthoppers at 4 d or 6 d post inoculation of ds*Translin*-RNA or ds*GFP*-RNA.


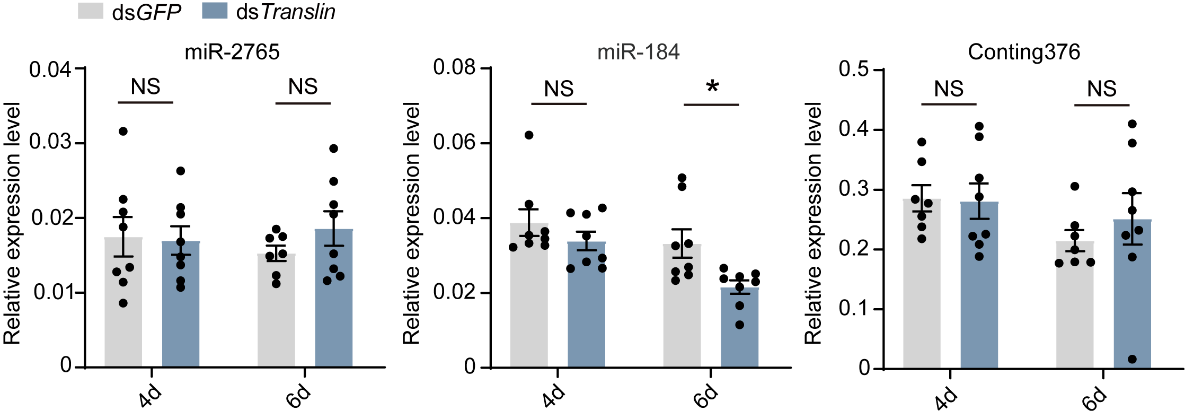


**Figure S3.** Verification of expression levels of several miRNAs using qPCR. Expression levels of three miRNAs relative to that of *U6* in viruliferous planthoppers at 4 d or 6 d post inoculation of ds*Translin*-RNA or ds*GFP*-RNA. n = 7-8 for each group. The values are presented as mean ± SE. Differences were statistically evaluated using Student’s *t*-test for comparison between two groups. NS, no significant differences. *, *p* < 0.05.


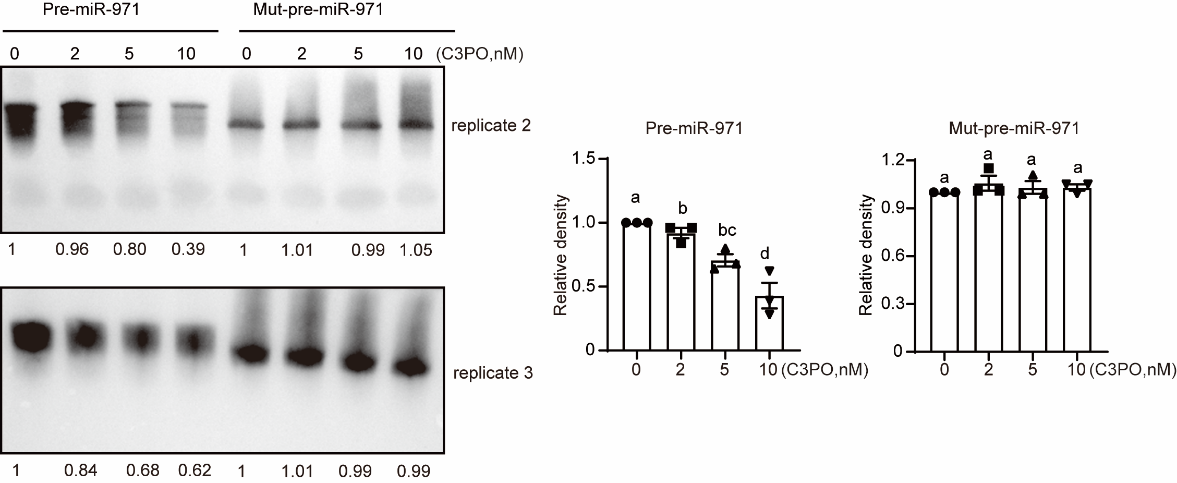


**Figure S4.** The direct degradation assay of C3PO to precursors of miR-971-3p, and mutants (Mut) labeled with biotin with two additional biological replicates. The purified products from pET28a vector serve as negative control. The relative grayscale values of miRNA precursors were shown underneath the photo, and statistically analyzed in GraphPad Prism 8.3.0. n = 3 for each group. Different letters on the columns represent significant differences. The values are presented as mean ± SE. Differences were statistically evaluated using one-way ANOVA followed by Tukey’s test for multiple comparisons.


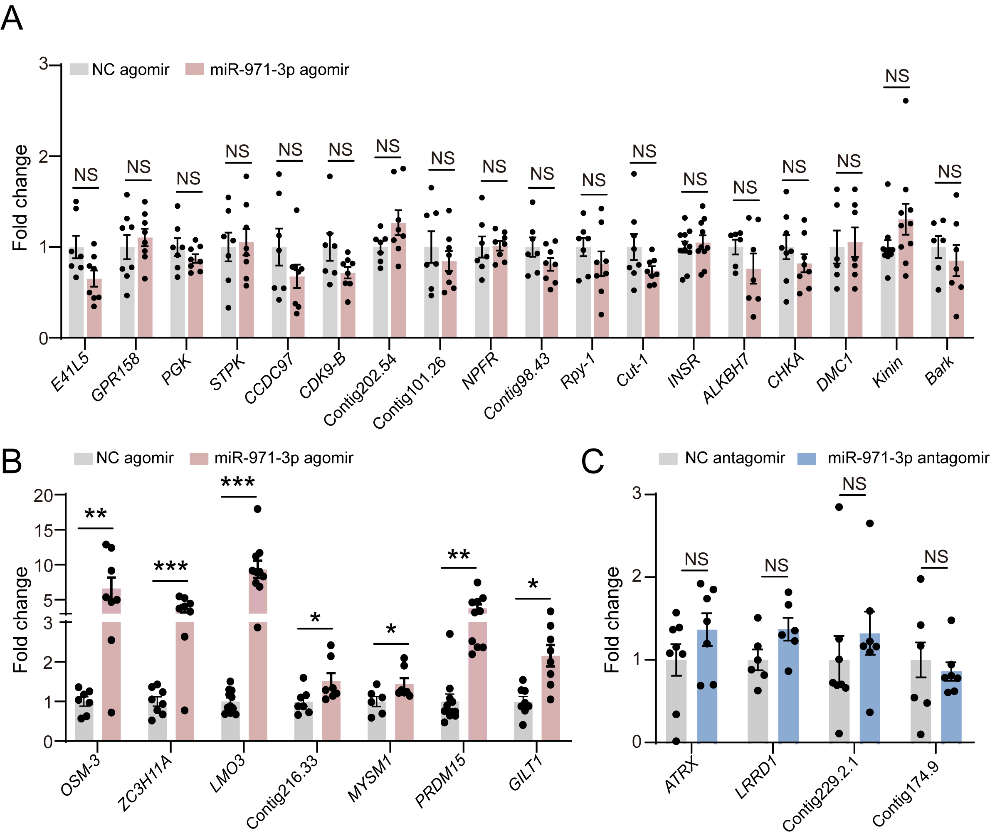


**Figure S5.** Fold change of transcript levels of miR-971-3p putative target genes in nonviruliferous planthoppers at 6 d after injection of miR-971-3p agomir (A, B) or antagomir (C) compared to those of injection with NC agomir or NC antagomir. n = 6-11 for each group. The values are presented as mean ± SE. Differences were statistically evaluated using Student’s *t*-test for comparison between two groups. NS, no significant differences. *, *p* < 0.05. **, *p*<0.01. ***, *p* < 0.001.


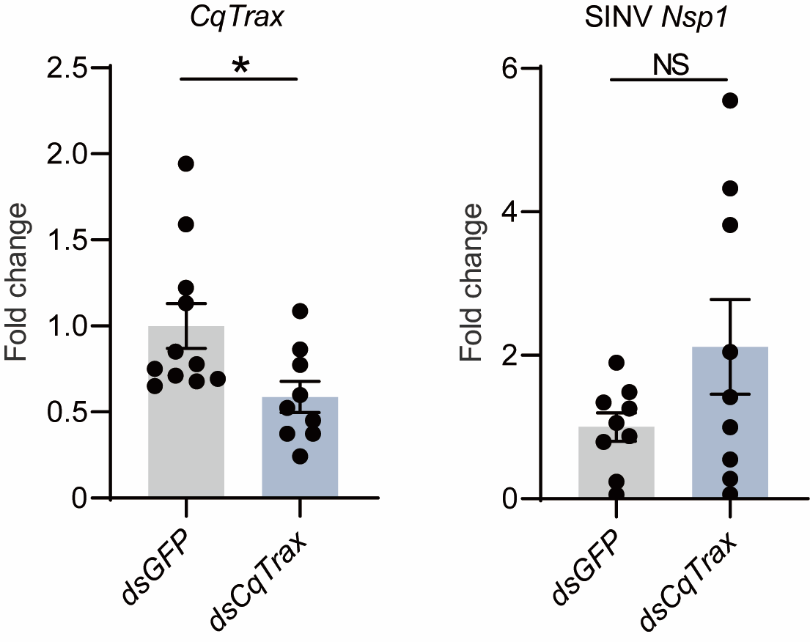


**Figure S6.** Fold change of *CqTrax* transcripts and RNA levels of SINV *Nsp1* in female mosquitoes at 4 d after injection of ds*CqTrax*-RNA and SINV virions compared to those of injection with ds*GFP*-RNA and virions. n = 9-11 for each group. The values are presented as mean ± SE. Differences were statistically evaluated using Student’s *t*-test for comparison between two groups. NS, no significant differences. *, *p*< 0.05.

Table S1. (separate file)

The expression levels of all planthopper microRNAs.

**Table S2.** Upregulated or downregulated miRNAs from small RNA-seq in viruliferous planthoppers at 4 d or 6 d post inoculation (dpi) of ds*Translin*-RNA

| miRNA | Sequence (5’-3’) | Dpi | Log_2_FC | Adjust *P* value |
| --- | --- | --- | --- | --- |
| Contig376 | CGGACGGAGAACUGAUAAGGGC | 6 | 6.3 | 3.72E-19 |
| Contig34128 | UGGGACUUCUAGCGGCAUAUGCC | 4 | 6.3 | 4.87E-39 |
| miR-2765 | CAAUGGUGCUGAAGUUCCUACA | 4 | 4.9 | 2.97E-08 |
| miR-971-3p | UUGGUGUUCUACCUUACAGUG | 6 | 4.2 | 1.90E-11 |
| Contig681/35303 | UGGUAACUACCAGCACAACCUCA | 4 | 3.0 | 0.02 |
| miR-87 | GUGAGCAAAGUUUCAGGUGUG | 4 | 2.3 | 6.10E-05 |
| Contig355_26279 | TGGGACTTCTAGCGGCATATGCC | 4 | -6.5 | 4.66E-37 |
| Contig1480_39226 | CGGACGGAGAACTGATAAGGGC | 6 | -6.2 | 8.56E-16 |
| Contig3502_41323* | CAATGGTGCTGAAGTTCCTACA | 4 | -4.5 | 5.05E-07 |
| miR-133a | TTTGGTCCCCTTCAACCAGCTG | 6 | -3.7 | 0.01 |
| Contig19158_44421* | TTGGTGTTCTACCTTACAGTG | 6 | -3.5 | 0.01 |
| Contig544-1 | UUAAAGCUUAUCCAACUGCGUA | 4 | -3.1 | 0.01 |
| miR-100-5p_1 | AACCCGTAGATCCGAACTTGTG | 6 | -3.0 | 0.01 |
| Contig122 | UAGGCUAGGCUGAUGAAUUCAA | 4 | -2.7 | 0.04 |
| Contig376_26626* | CCTTATCATTCTTCTGTCGGG | 4 | -2.3 | 1E-03 |

FC, fold change

Table S3. (separate file)

The candidate target genes of miR-971-3p predicted by both miRanda and RNAhybrid algorithms in planthoppers.

**Table S4.** Primers used in this study

| **Primer name** | **Primer sequence (5’ to 3’)** |
| --- | --- |
| **primers used for qPCR** | |
| Contig34128-qPCR-F | TGGGACTTCTAGCGGCATATGCCAAA |
| miR-2765-qPCR-F | GCGCCAATGGTGCTGAAGTTCCTACAAA |
| miR-184-qPCR-F | GCGCCCTTATCATTCTTCTGTCGGGAA |
| miR-87-qPCR-F | GCGCGTGAGCAAAGTTTCAGGTGTGAA |
| Contig681/35303-qPCR-F | TGGTAACTACCAGCACAACCTCA |
| Contig376-qPCR-F | CGGACGGAGAACTGATAAGGGCAA |
| miR-971-3p-qPCR-F | GCGCTTGGTGTTCTACCTTACAGTGAA |
| Pre-miR-971-3p-qPCR-F | CGACGTTTGGTGTTCTACCTTACA |
| *U6*-qPCR-F | TGGAACGATACAGAGAAGATTAGCA |
| *U6*-qPCR-R | AACGCTTCACGATTTTGCGT |
| *NHLRC2*-qPCR-F | GGACGGGCTAGTTGTGGTT |
| *NHLRC2*-qPCR-R | CAGCGTGCGAGATGTGATAG |
| *NP*-qPCR-F | GGAACAAATGCCAATGCTATC |
| *NP*-qPCR-R | TGAGACATTTGGGAATAGCTGA |
| *EF2*-qPCR-F | GTCTCCACGGATGGGCTTT |
| *EF2*-qPCR-R | ATCTTGAATTTCTCGGCATACATTT |
| *Translin*-qPCR-F | TGAAGAATGATGACGAGCGTTT |
| *Translin*-qPCR-R | TCACGAGCCTTCGAGCAATA |
| *Trax*-qPCR-F | CGGACGATATTGTTGAGGACTC |
| *Trax*-qPCR-R | TTGAGGCATCAGTGGAGACC |
| *HlTranslin*-qPCR-R | AGGCTGTCGTTCTTGAGGTT |
| *MYSM1*-qPCR-F | GCCAAGAGTTGGTAATGGTGTA |
| *MYSM1*-qPCR-R | CCGTGGTATCTCTCTGCTGAT |
| *LMO3*-qPCR-F | CACCATCATCACCACCATCAC |
| *LMO3*-qPCR-R | ACACTTTAACAGCGGCACAAT |
| *CHKA*-qPCR-F | CGAACATTTGCTCATGGAAGTC |
| *CHKA*-qPCR-R | CGAACGGTATCTGCGAAGTC |
| *CDK9-B*-qPCR-F | TTGAATCAGCATCCAAGACAGT |
| *CDK9-B*-qPCR-R | CAATGCACACCAGTCACCAT |
| *Kinin*-qPCR-F | GAGTGAGTCTGATGGAAGCAAT |
| *Kinin*-qPCR-R | ACGTTACTGTGTTGGTTGGAA |
| *GILT1*-qPCR-F | AGTGCAGACAATACACGTTTGA |
| *GILT1*-qPCR-R | TGGCTTTATCCCTCGTTGATTC |
| *PRDM15*-qPCR-F | AATAGTGGCGGTCAGACAGT |
| *PRDM15*-qPCR-R | TTGAGGCAGCAGAGGTTCC |
| *ZC3H11A*-qPCR-F | ATCGGCAGATTCTTGATGTACC |
| *ZC3H11A*-qPCR-R | GCTTGTCTGACCATCTTCGG |
| *LRRD1*-qPCR-F | AGCTGTTTCTCACAAGGGTTT |
| *LRRD1*-qPCR-R | GTTGCTCCTCAGGTCCAGAT |
| *Cut-1*-qPCR-F | ACCAATGTAATGTGCGTCTCTG |
| *Cut-1*-qPCR-R | TTCCTTCGTCGTCGTCCTG |
| *INSR*-qPCR-F | TTGAATGTTGACAGCATCTCCT |
| *INSR*-qPCR-R | CAGAGGGAAAGAACGCACAA |
| *OSM*-3-qPCR-F | TGGCGTCTACTACATCAACTCA |
| *OSM*-3-qPCR-R | AGGCGAACACCGTGCTATT |
| *E41L5*-qPCR-F | CGGTCAGAAGGCTACTCACA |
| *E41L5*-qPCR-R | TCCACAGATAAGTCGGTTCCAT |
| *CCDC97*-qPCR-F | GCAAGACAATCTCTACGTGATG |
| *CCDC97*-qPCR-R | GCCTCTTCAAGTTCAGGTTCT |
| Contig174.9-qPCR-F | TCCTCATCATCTTCCTCCAGTT |
| Contig174.9-qPCR-R | AGCCGCTACATTGTAAGCATAG |
| *ATRX*-qPCR-F | AACATCAATGGCGTACCTCAAG |
| *ATRX*-qPCR-R | GATTCAACACGAACCACTTCCA |
| Conting216.33-qPCR-F | TCATATCGTGTTTGCACTTGTG |
| Conting216.33-qPCR-R | CTGGAACCTCGAAGCTAAGAG |
| *Rpy*-1-qPCR-F | CGGCTCTACAACCAACACAAG |
| *Rpy*-1-qPCR-R | GATTCGGACTGTCTGCTTCTTC |
| Conting202.54-qPCR-F | TTCTCTCTGCTTTCCGGTCC |
| Conting202.54-qPCR-R | GAACGAAGAAGTGCGGTCTG |
| *ALKBH7*-qPCR-F | GCCTCAGTCTTCTATCGGATTC |
| *ALKBH7*-qPCR-R | CGGTAATATCTCGTGCGTGAA |
| *DMC1*-qPCR-F | AATGGTTCTAAGCGAGATGACC |
| *DMC1*-qPCR-R | GCACATCACAATTCCTTTGACT |
| Contig101.26-qPCR-F | GGCTTCAGGAAGTCGCAATAC |
| Contig101.26-qPCR-R | TCAGCATCTGTGAAACCCATCT |
| *Bark*-qPCR-F | AACCGTAGTTGCTTACAGTGAA |
| *Bark*-qPCR-R | TGTTGTGTCCGCATTCAGC |
| *NPFR*-qPCR-F | ACATCAGGAGTTGTGTCCAATG |
| *NPFR*-qPCR-R | CAGCGAGATCAGCGTGAAC |
| Contig98.43-qPCR-F | TTGCCTCCTGCTGAAGACA |
| Contig98.43-qPCR-R | ACGAACTGCTCACAAGATAAGA |
| Contig229.2.1-qPCR-F | GCTTGTAACCGTTGACTGGAG |
| Contig229.2.1-qPCR-R | GACTGGAGCATCGCTTGTG |
| *PGK*-qPCR-F | AGAAGACGGCTTCGGGAAT |
| *PGK*-qPCR-R | TGGAGTGTCTTTGTGAACTTGA |
| *STPK*-qPCR-F | CTCTTCACTCTTGTGTGATCTT |
| *STPK*-qPCR-R | CTTATAAAGCGATTCGCTGACA |
| *GPR158*-qPCR-F | ACATAACAGAAGCAGGTGAACT |
| *GPR158*-qPCR-R | GCAGTGTTTCCCAGTAGAGAG |
| RIP-*NHLRC2*-F | TGGAAGAACAGATCGTACACTC |
| RIP-*NHLRC2*-R | CGACATCGGGTACAGGTTG |
| SFTSV *Gc*-qPCR-F | GATGAGATGGTCCATGCTGATTCT |
| SFTSV *Gc*-qPCR-R | CTCATGGGGTGGAATGTCCTCAC |
| *ELFA*-qPCR-F | CGTCTACAAGATTGGTGGCATT |
| *ELFA*-qPCR-R | CTCAGTGGTCAGGTTGGCAG |
| *CqTranslin*-qPCR-F | ACCTGGAGAAGGGATTCCTG |
| *CqTranslin*-qPCR-R  *CqTrax*-qPCR-F  *CqTrax*-qPCR-R | ACTCCCATCAGGTAGTCCTC  GGTTCATGCAGGAGCTGTAC  CACGGACCTTCACGTTGTAG |
| SINV *Nsp1*-qPCR-F | GAGGTAGTAGCACAGCAGG |
| SINV *Nsp1*-qPCR-R | CGGAAAACATTCTACGAGC |
| *β-actin*-qPCR-F | CGGGTATTGTGCTGGACTC |
| *β-actin*-qPCR-R | GCGACGTAGCACAGCTTCT |
| **Primers used for RNAi** | |
| *GFP*-RNAi-F | CACAAGTTCAGCGTGTCCG |
| *GFP*-RNAi-R | GTTCACCTTGATGCCGTTC |
| *GFP*-RNAi-T7F | TAATACGACTCACTATAGGCACAAGTTCAGCGTGTCCG |
| *GFP*-RNAi-T7R | TAATACGACTCACTATAGGGTTCACCTTGATGCCGTTC |
| *Translin*-RNAi-F | TGCTCGAAGGCTCGTGAA |
| *Translin*-RNAi-R | ATTCTCCGTTGAAGTCAAGTGT |
| *Translin*-RNAi-T7F | TAATACGACTCACTATAGGTGCTCGAAGGCTCGTGAA |
| *Translin*-RNAi-T7R | TAATACGACTCACTATAGGATTCTCCGTTGAAGTCAAGTGT |
| *Trax*-RNAi-F | GAGTCGGGTACAATCTGTTCAT |
| *Trax*-RNAi-R | ACCAACAAGTCCTGCGAAAC |
| *Trax*-RNAi-T7F | TAATACGACTCACTATAGGGAGTCGGGTACAATCTGTTCAT |
| *Trax*-RNAi-T7R | TAATACGACTCACTATAGGACCAACAAGTCCTGCGAAAC |
| *NHLRC2*-RNAi-F | GCCGACTCCTACAATCACAAG |
| *NHLRC2*-RNAi-R | AGTTGCCATTCTGCTGTTGAG |
| *NHLRC2*-RNAi-T7F | TAATACGACTCACTATAGGGCCGACTCCTACAATCACAAG |
| *NHLRC2*-RNAi-T7R | TAATACGACTCACTATAGGAGTTGCCATTCTGCTGTTGAG |
| *HlTranslin*-RNAi-F | TGTCATTCCAGCAGCACTTG |
| *HlTranslin*-RNAi-R | TCTCCAGGAACACGGTCAG |
| *HlTranslin*-RNAi-T7F | TAATACGACTCACTATAGGTGTCATTCCAGCAGCACTTG |
| *HlTranslin*-RNAi-T7R | TAATACGACTCACTATAGGTCTCCAGGAACACGGTCAG |
| *CqTranslin*-RNAi-F | TCCGAACTGAGCCGTTACGC |
| *CqTranslin*-RNAi-R | CACGAATGCTGATGTCGTAC |
| *CqTranslin*-RNAi-T7F | TAATACGACTCACTATAGGNTCCGAACTGAGCCGTTACGC |
| *CqTranslin*-RNAi-T7R | TAATACGACTCACTATAGGNCACGAATGCTGATGTCGTAC |
| *CqTrax*-RNAi-F  *CqTrax*-RNAi-R | TGTGCGAGGAAGCGAAGAA  GGATCATCCGTCTCCATCGT |
| *CqTrax*-RNAi-T7F | TAATACGACTCACTATAGGTGTGCGAGGAAGCGAAGAA |
| *CqTrax*-RNAi-T7R | TAATACGACTCACTATAGGGGATCATCCGTCTCCATCGT |
| **Primers used for expression plasmid construction** | |
| SINV *Nsp1*-F | TCACAATGGAGAAGCCAGTAGT |
| SINV *Nsp1*-R | ACGGTCATTTGCTTGAGGTATT |
| SINV *Nsp1*-NcoI-F | AGGAGATATACCATGGGCATGGAGAAGCCAGTA |
| SINV *Nsp1*-XhoI-R | GGTGGTGGTGCTCGAGTGCTCCGATGTCCGC |
| SINV *Nsp2*-F | GGACATCGGAGCAGCATTAGT |
| SINV *Nsp2*-R | TGCGTTGACAACTGCTTCCT |
| SINV *Nsp2*-NcoI-F | AGGAGATATACCATGGGCATGGCATTAGTCGAAACCC |
| SINV *Nsp2*-XhoI-R | GGTGGTGGTGCTCGAGGGCTCCAACTCCGTCTC |
| SINV *Nsp3*-F | GGTACAAGAGATGGAGTTGGAG |
| SINV *Nsp3*-R | CGGTTAGTCAGTATTCAGTCCT |
| SINV *Nsp3*-NcoI-F | AGGAGATATACCATGGGCATGGCACCGTCGTACCGT |
| SINV *Nsp3*-XhoI-R | GGTGGTGGTGCTCGAGGTATTCAGTCCTCCTG |
| SINV *RdRp*-F | ATGCTAACCGGGGTAGGTGGGTAC |
| SINV *RdRp*-R | TTTAGGACCACCGTAGAGATGCTT |
| SINV *RdRp*-NcoI-F | AGGAGATATACCATGGGCATGATGCCCACCGAAGC |
| SINV *RdRp*-XhoI-R | GGTGGTGGTGCTCGAGTTTAGGACCACCGTAGAGATG |
| *CqTranslin*-F | ATGCAGAACGCGGTGATTAAGGAC |
| *CqTranslin*-R | TCACTGCTCGGAAGCAGCAG |
| *CqTranslin*-SmaI-F | GGTCGTGGGATCCCCGGGATGCAGAACGCGGTGATTAA |
| *CqTranslin*-SmaI-R | TCACGATGAATTCCCGGGTCACTGCTCGGAAGCAGCG |
| 5’ RACE-*HlTranslin*-F | AGGTCGTAGACCACCTCCTCGACCTTC |
| 3’ RACE-*HlTranslin*-F | ACCGAGATGAACCTGGGCTTCCGGCT |
| *Translin*-F | ATGGATTTAGATTTTAAATCG |
| *Translin*-R | CTAACTCTTATTCTCCGTTG |
| *Translin*-NcoI-F | AGGAGATATACCATGGGCATGGATTTAGATTTT |
| *Translin*-XhoI-R | GGTGGTGGTGCTCGAGACTCTTATTCTCC |
| *Translin*-SmaI-F | GGTCGTGGGATCCCCGGGATGGATTTAGATTTT |
| *Translin*-SmaI-R | TCACGATGAATTCCCGGGACTCTTATTCTCC |
| RSV *RdRp2*-NcoI-F | AGGAGATATACCATGGGCGAGGCTGTTGATGTAA |
| RSV *RdRp2*-XhoI-R | GGTGGTGGTGCTCGAGTGATTCTGGACTGCC |
| *Trax*-F | CAAAGGTGACTGAAATCAGACA |
| *Trax*-R | AGGCTAATCGTAACTACATTCC |
| *Trax*-EcoRI-F | TCGCGGATCCGAATTCATGGCAGACC |
| *Trax*-HindIII-R | GTGCGGCCGCAAGCTTATCGTAACTA |
| Rluc-*NHLRC2*-F | ATTTCCTCGCCGTGGGACGT |
| Rluc-*NHLRC2*-R | ACCGTCTTGCAGTTGCAGTCG |
| Rluc-*NHLRC2*-XhoI-F | TAGGCGATCGCTCGAGATTTCCTCGCCGTGGGACGT |
| Rluc-*NHLRC2*-NotI-R | TTGCGGCCAGCGGCCGCACCGTCTTGCAGTTGCAGTCG |
| Mut-Rluc-*NHLRC2*-F | CCACAACACAACCTGTACCCGATGTCGGCGTCTTTTGCG |
| Mut-Rluc-*NHLRC2*-R | CTCCTCCTTGCCGTTTCCAGCCACGCATGCGCAC |
| *NHLRC2*-F | ATGGCGTTCGATTTTCACGATATTCAA |
| *NHLRC2*-R | CTATAACTTAATAGTATGCTTAATATCACAAA |
| *NHLRC2*-NcoI-F | AGGAGATATACCATGGGCATGGCGTTCGATTTTCACGATATTCAA |
| *NHLRC2*-XhoI-R | GGTGGTGGTGCTCGAGTAACTTAATAGTATGCTTAATATCACAAA |
| RSV *RdRp2*-mut-D547-F | GCGAAGAGCTTTAAGGGTGCTGACTTATCT |
| RSV *RdRp2*-mut-D547-R | AGTCTCAGTTGATAAGATATCAAAAACAAA |
| RSV *RdRp2*-mut-D567-F | GCGGTCATTATATCAAGAGAAAGTGATCCAC |
| RSV *RdRp2*-mut-D567-R | TGGTGACCAGTTATCTGAGATTCCTCC |
| RSV *RdRp2*-mut-E585-F | GCCTTTACAACTAGGTCCACTGAGTCTATAG |
| RSV *RdRp2*-mut-E585-R | ATAGACAACGATATCTTCATACTGTGGATC |
| RSV *RdRp2*-mut-K604-F | GCGAGCTTACGATATAAAGAAGCAATTCAGG |
| RSV *RdRp2*-mut-K604-R | AACCTCTACTGATCTTAGTAGAGATTC |

F, forward primer; R, reverse primer.
